# Microbial diversity gradients in the geothermal mud volcano underlying the hypersaline Urania Basin

**DOI:** 10.3389/fmicb.2022.1043414

**Published:** 2022-12-21

**Authors:** Cassandre Sara Lazar, Frauke Schmidt, Marcus Elvert, Verena B. Heuer, Kai-Uwe Hinrichs, Andreas P. Teske

**Affiliations:** ^1^Department of Biological Sciences, Université du Québec à Montréal, Montréal, QC, Canada; ^2^Organic Geochemistry Group, Department of Geosciences, MARUM Center for Marine Environmental Sciences, University of Bremen, Bremen, Germany; ^3^Department of Earth, Marine and Environmental Sciences, University of North Carolina at Chapel Hill, Chapel Hill, NC, United States

**Keywords:** archaea, bacteria, hypersaline basin, cold seep, marine subsurface sediments, Urania Basin

## Abstract

Mud volcanoes transport deep fluidized sediment and their microbial communities and thus provide a window into the deep biosphere. However, mud volcanoes are commonly sampled at the surface and not probed at greater depths, with the consequence that their internal geochemistry and microbiology remain hidden from view. Urania Basin, a hypersaline seafloor basin in the Mediterranean, harbors a mud volcano that erupts fluidized mud into the brine. The vertical mud pipe was amenable to shipboard Niskin bottle and multicorer sampling and provided an opportunity to investigate the downward sequence of bacterial and archaeal communities of the Urania Basin brine, fluid mud layers and consolidated subsurface sediments using 16S rRNA gene sequencing. These microbial communities show characteristic, habitat-related trends as they change throughout the sample series, from extremely halophilic bacteria (KB1) and archaea (*Halodesulfoarchaeum* spp.) in the brine, toward moderately halophilic and thermophilic endospore-forming bacteria and uncultured archaeal lineages in the mud fluid, and finally ending in aromatics-oxidizing bacteria, uncultured spore formers, and heterotrophic subsurface archaea (Thermoplasmatales, Bathyarchaeota, and Lokiarcheota) in the deep subsurface sediment at the bottom of the mud volcano. Since these bacterial and archaeal lineages are mostly anaerobic heterotrophic fermenters, the microbial ecosystem in the brine and fluidized mud functions as a layered fermenter for the degradation of sedimentary biomass and hydrocarbons. By spreading spore-forming, thermophilic Firmicutes during eruptions, the Urania Basin mud volcano likely functions as a source of endospores that occur widely in cold seafloor sediments.

## 1 Introduction

The Urania Basin is situated southwest off the coast of Crete, in the inner plateau of the Mediterranean Ridge characterized by the presence of Messinian salt evaporites within the sediments, hundreds of meters below the seafloor (Westbrook and Reston, 2002). With a chloride content of 120 g/L, i.e., more than five times of that in Mediterranean seawater, Urania Basin brine is the least saline of the Mediterranean DHABs (deep hypersaline anoxic basin); yet Urania Basin brine contains exceptionally high levels of methane (up to 5.56 mM) and sulfide (up to 16 mM) (Borin et al., 2009), making Urania Basin one of the most sulfidic marine water bodies on Earth (Karisiddaiah, 2000; Charlou et al., 2003). Despite these extreme conditions, active microbial communities are present (van der Wielen et al., 2005) and contribute to biogeochemical cycling of carbon and sulfur in the deep anoxic hypersaline brine of the Urania Basin.

A unique feature of the Urania Basin is the presence of an active mud volcano in the southwestern part of the horseshoe-shaped basin, found by a CTD survey (conductivity, temperature, depth) during the R/V Urania expedition that discovered the basin in 1993. Elevated temperatures of up to 55°C coincide with active mud volcanism underneath the brine layer (Corselli et al., 1996; Fusi et al., 1996). A direct association between brines and active mud volcanism has been previously documented by exotic deposits sampled in the outer rim of the basin (Hübner et al., 2003) and by the presence of sediments containing Plio-Pleistocene microfossils at the bottom of the basin (Aloisi et al., 2006). The fluidized mud volcano sediment, consisting of micrometer-sized particles, nannofossils, carbonate and gypsum grains, constitutes a convectively mixed suspended seafloor that extends vertically over 110 m before transitioning into solidified subsurface sediment (Aiello et al., 2020).

The Urania Basin mud volcano provides a valuable model system to test the working hypothesis that mud volcanoes bring deep subsurface microorganisms to the surface and distribute them across the deep-sea floor. As conduits of fluid migration from the deep subsurface to the seafloor, mud volcanoes provide windows into the deep subseafloor biosphere (Hoshino et al., 2017) that is otherwise only accessible by deep drilling. Since the chemistry of the discharging fluidized muds varies within and between sites, different combinations of brine, methane, gaseous and liquid hydrocarbons, sulfides, nutrients and low-molecular weight organic acids shape the resident microbial communities and their activities, as shown in numerous individual studies (Joye et al., 2009; Pachiadaki et al., 2010, 2011; Lazar et al., 2011a,b, 2012; Pachiadaki and Kormas, 2013). Mud volcanoes are pulsating with fluidized mud that frequently overflows their surrounding seafloor basins (McDonald et al., 2000; Hübner et al., 2003; Teske and Joye, 2020) and by the same token deposit fluid mud-hosted bacteria and archaea. Mud volcano and cold seep geofluids have been proposed as the carrier medium for seeding surficial seafloor sediments with endospore-forming thermophilic Firmicutes that could not grow under cold *in situ* conditions (Hubert et al., 2009; Rattray et al., 2022).

To explore the spatially shifting microbial community in the Urania Basin brine/mud volcano system, we analyzed bacterial and archaeal 16S rRNA gene amplicons along a vertical profile from consolidated subseafloor sediments at the underlying seabed at the bottom of the mud volcano (the “pit”), through fluidized mud volcano sediment layers that extend from the subsurface seabed to the bottom of the brine lake, to the overlying brine lake. This approach was supported by analysis of gaseous hydrocarbons and hydrocarbon lipid biomarkers from selected sediment samples. A previous 16S rRNA gene sequencing survey of deep mud fluid sediment has uncovered diverse bacterial and archaeal populations (Yakimov et al., 2007) but did not produce a microbial profile through all three layers (subsurface sediment, fluid mud layers, brine fluid). The geochemical and microbiological changes observed along this depth profile indicate that each sediment and brine layer has selected for its unique microbial community.

## 2 Materials and methods

### 2.1 Sediment sampling

**FIGURE 1:**
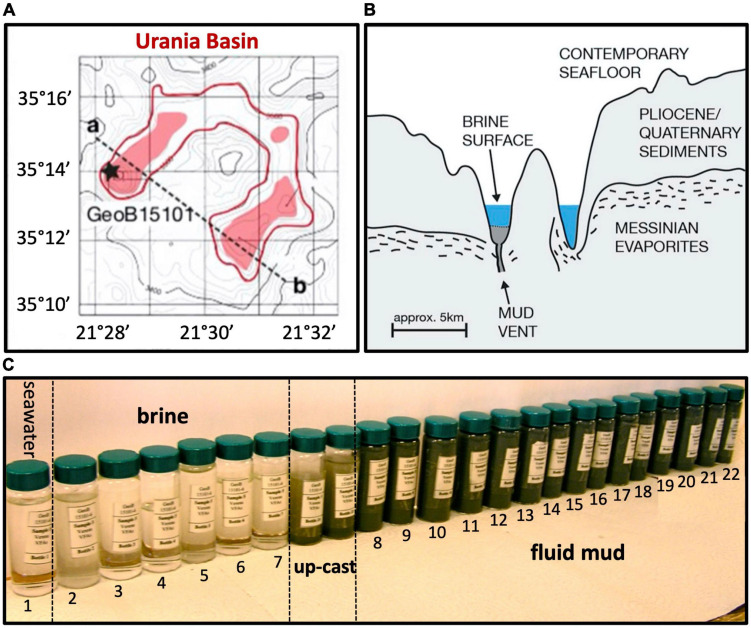
**(A)** Lower left, bathymetry of Urania Basin with bottom brines highlighted in red, and profile line drawn from the mud volcano in the northwestern end toward the southeastern end of Urania Basin. **(B)** Geologic sketch of Urania Basin, following the profile line between points a and b. **(C)** Sample vials with Niskin bottle samples from seawater, brine, and fluidized mud. The samples between brine and mud layer represent perturbed samples from the CTD upcast (GeoB15101-6, Zabel, 2012) at 3,605 and 3,610 m depth. All figure panels modified from Aiello et al. (2020).

Brine, mud fluids and subseafloor sediments were sampled in the Urania Basin during cruise M84/1 (DARCSEAS) of R/V Meteor in February 2011. Two CTD casts GeoB15101-1 and GeoB15101-6 (35°13.86′N, 21°28.30′E) deployed twenty-four Niskin bottles each at increasing water depths, and samples were used for stable ion measurements (Aiello et al., 2020) and DNA extractions, respectively. Based on stable ion concentrations and CTD pressure readings, the seawater-brine interface was located between 3,461 and 3,463 m water depth (mwd), and the brine/fluid mud interface between 3,607 and 3,610 m water depth (Aiello et al., 2020). Within the fluid mud layers, sampling depths were conservatively based on CTD cable length since the high density of the mud interfered with reliable pressure measurements by the CTD sensors as soon as the CTD entered the fluid mud layer. For GeoB15101-1, bottles 1 (70 m water depth, mwd) to 8 (3,461 mwd) sampled the overlying seawater, whereas bottles 9 (3,463 mwd) to 24 (3,598 mwd) sampled the hypersaline brine lake. For cast GeoB15101-6 (used in this study), bottle 1 sampled overlying seawater (3,000 mwd), and bottles 2 (3,560 mwd) to 7 (3,605 mwd) sampled the hypersaline brine. The underlying mud fluid layers were sampled in 10-m intervals by bottles 8 (3,610 m cable length) to 20 (3,730 m cable length). The deepest mud fluid sample, no. 21, was collected at a cable length of 3,736 m when the sediment became sufficiently consolidated to prevent further sinking of the CTD. Subsamples taken from the 24 Niskin bottles of the CTD cast GeoB15101-6—sampled progressively from the seawater, through the Urania brine lake, down to the seafloor—clearly demonstrated the fluid mud layer starting with bottle 8, and continuing downcore toward gradually darker mud (Figure 1). The bottom of the fluidized mud layer (sample 21, 3,736 m cable length) was 110 m below the expected seafloor depth, where brine transitions into mud, based on echo-sounder data (Zabel, 2012).

The subsequently deployed gravity core GeoB15101-7 (35°13.87′N, 21°28.30′E) recovered 4.5 m of the consolidated sediments below the mud fluid layers, and depths for these sediment samples are given in cm from the top of the core. Subsamples of each Niskin bottle of cast GeoB15101-6 as well as selected gravity core sections of cast GeoB15101-7 were immediately stored at –20°C for subsequent molecular analyses, including hydrocarbon lipid biomarkers. Concentration measurements of methane and ethane were carried out onboard the ship, whereas investigation of the gaseous hydrocarbon’s stable carbon isotopes was performed in the home lab.

### 2.2 Hydrocarbon measurements

Concentrations of dissolved methane and ethane were determined according to previously reported protocols (Pimmel and Claypool, 2001; D’Hondt et al., 2003), using two to three mL of brine, mud fluid and subseafloor sediment samples (see Supplementary Methods for details). Isotope values of methane and ethane are reported in the delta notation (δ^13^C) relative to the Vienna Pee Dee Belemnite (VPDB) standard using a calibrated in-house CO_2_ reference gas standard. For hydrocarbon lipid biomarker analysis of two gravity core sediments samples from 10 to 30 and 260–280 cm core depth, 50 g of freeze-dried sample material was extracted using a modified Bligh and Dyer protocol (Sturt et al., 2004). Hydrocarbons were identified *via* their known mass spectra and concentrations were calculated from their peak area in the FID chromatogram relative to the injection standard (see Supplementary Methods for details).

### 2.3 DNA extraction, PCR amplification and 16S rRNA gene library construction

Samples chosen for molecular analyses included Niskin bottles 5 (3,590 mwd, hypersaline brine), 8 (3,610 m, top of the mud fluid layer), 14 (3,670 m, middle of the mud fluid layer) and 21 (3,736 m, bottom of the mud fluid layer) from the GeoB15101-6 CTD cast (Figure 1), and depth sections 10–30 and 260–280 cmbsf of the GeoB15101-7 gravity core. DNA was extracted using a bead beating soil extraction kit on 10 g of wet sediment for the mud fluid and gravity core samples (UltraClean^®^ Mega Soil DNA Isolation Kit, MO BIO, CA now QIAGEN), and using 50 mL of filtered brine (PowerSoil^®^ DNA Isolation Kit, Mo BIO, CA now QIAGEN). The extracted DNA was then purified using the Wizard DNA Clean-Up System (Promega, Wisconsin, USA).

Archaeal 16S rRNA genes were amplified using the primers A24F (5′-CGGTTGATCCTGCCGGA-3′) and A1492R (5′-GGCTACCTTGTTACGACTT-3′) (Casamayor et al., 2000) and the Flexi GoTaq DNA polymerase (Promega, WI). A total of 35 PCR cycles (1 min denaturation at 94°C, 1′30 min annealing at 50°C, and 2 min elongation at 72°C) were run in a BIO-RAD iCycler (Hercules, CA). A nested PCR using the primer set A24F (5′-CGGTTGATCCTGCCGGA-3′; Casamayor et al., 2000) and Arch915R (5′-GTGCTCCCCCGCCAATTCCT-3′, Stahl and Amann, 1991) was necessary in order to obtain sufficient amounts of archaeal 16S rRNA gene amplicons for cloning, using the following conditions: 1 min denaturation at 94°C, 1′30 min annealing at 58°C, and 2 min elongation at 72°C, repeated for 35 cycles.

Bacterial 16S rRNA genes were amplified using the B8F-B1492R primer set (5′-AGRGTTTGATCCTGGCTCAG-3 and 5′-CGGCTACCTTGTTACGACTT-3; Teske et al., 2002) using the following PCR conditions: 1 min denaturation at 94°C, 1′30 min annealing at 50°C, and 2 min elongation at 72°C, repeated for 26 cycles to limit contamination since the extracted DNA concentrations were extremely low. A nested PCR using the primer combination B27F (5′-AGA GTTTGATCCTGGCTCAG-3′) and U1406R (5′-GACGGGC GGTGTGTRCA-3′; Heuer et al., 1997) was necessary to obtain sufficient amplicon material for cloning, using the following conditions: 1 min denaturation at 94°C, 1′30 min annealing at 50°C, and 2 min elongation at 72°C, repeated for 30 cycles. PCR products were purified using the QIAquick Gel Extraction Kit (QIAGEN, CA, USA) prior to cloning, and were then cloned using the TOPO XL cloning kit (Invitrogen, CA). After inserting the PCR amplified genes into the provided plasmid, the plasmid DNA was incorporated into *Escherichia coli* TOPO10 One Shot cells. Transformed cells were grown overnight, and colonies were collected for Sanger sequencing.

### 2.4 DNA sequencing and phylogenetic analysis

The microbial census of the Urania Basin mud layers is deliberately based on nearly full-length 16S rRNA gene clone libraries for Bacteria (1,379 bp in *E. coli* standard notation) and widely used large fragments for archaea (890 bp for the Archaea), in order not to compromise the phylogenetic placement and taxonomic identification of potentially novel microbial populations in this unusual extreme habitat, and to provide a well-curated reference dataset for 16S-rRNA based comparison that is not limited by sequence length. Furthermore, given the nature of the extremely hypersaline samples, DNA analyses *via* high-throughput sequencing revealed themselves to be ineffective by only yielding a dozen reads.

Sequences were obtained by Genewiz (South Plainfield, NJ) on an ABI Prism 3730xl sequencer using the M13R primer. Chimeras were identified with Mothur’s chimera slayer feature (Schloss et al., 2009), complemented by Blast analysis of sequence segments, and removed from phylogeny inferences. For initial phylogenetic analyses, the 16S rRNA gene sequences were edited in the BioEdit v7.0.5 program (Hall, 1999) and aligned by using the SINA webaligner (Pruesse et al., 2012).^1^ The alignment was then manually checked using ARB (Ludwig et al., 2004) and initial phylogenetic trees were calculated using the neighbor-joining method in ARB. Robustness of the inferred topology was tested by bootstrap resampling with 1,000 replicates. Final phylogeny figures were constructed with sequences selected from cultured bacteria and archaea and complemented by environmental sequences (limited to *ca*. 50–80 sequences for readability), and manually aligned for secondary rRNA structure to ensure consistent comparisons of homologous nucleotide positions across variable and hypervariable regions (Schloss, 2013); subsequently, tree topology was checked by 1,000 bootstrap replicates (Swofford, 2000). Whenever possible, pure-culture isolates with close sequence similarity to Urania Basin clones (for genus level > 95%, Schloss and Handelsman, 2005) were preferred for phylogenetic comparisons, to limit the inherent uncertainties of sequence-based physiological inferences (see Langille et al., 2013 for discussion). For uncultured lineages, Urania Basin sequences were identified as specifically as possible by comparisons to published subgroup analyses and phylogenies, with metagenomes when possible. The sequence data reported here were submitted to the GenBank nucleotide sequence database under the accession numbers OP352539-OP352606, OP352609-OP352677, OP352609-OP352677, OP352680-OP352749, OP352780-OP352844, OP389299-OP389989, and OP856603-OP856629.

## 3 Results

### 3.1 Depth stratification and biogeochemistry

During CTD deployment, a prominent halocline was detected at about 3,465 mwd, and corresponded to a salinity increase from ∼39 to ∼152 PSU (practical salinity unit), a 2.5°C increase in temperature (14–16.5°C) and the complete depletion of free oxygen; this transition marks the seawater-brine interface (Zabel, 2012). Once the CTD cast went through the brine layer and reached the depth of the seafloor, identified by onboard echo sounder at approximately 3,600 m, the cable tension did not drop, and the deployment was continued below that depth. About 160 m within the brine, at 3,625 mwd, a second density boundary was detected below which the CTD malfunctioned, and from then on depth was measured in cable length. The deployment continued until 3,735 m cable length, when a relatively fast decrease of the cable tension indicated a third boundary as the CTD rosette reached sufficiently consolidated sediments that stopped further sinking. This depth was 110 m below the expected seafloor depth based on echo sounder data (Zabel, 2012).

**FIGURE 2:**
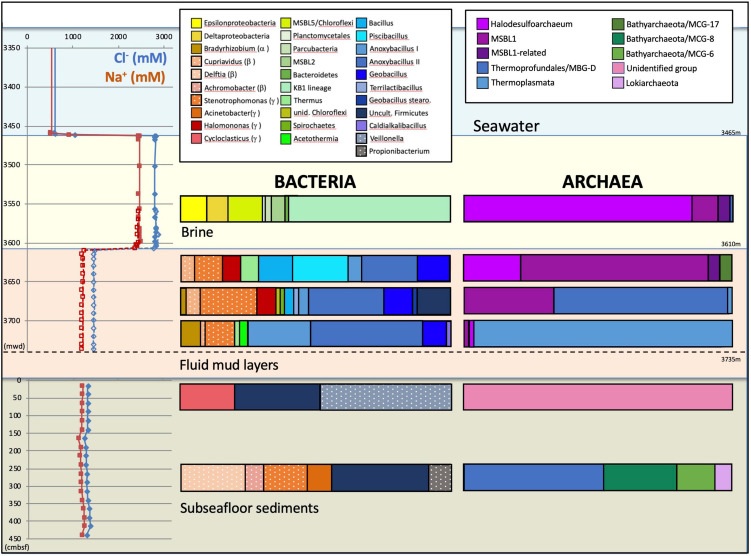
Depth profiles of water and sediment porewater geochemistry and phylogenetic affiliations of the archaeal and bacterial 16S rRNA gene sequences for the three Urania Basin habitats: subseafloor sediments, mud fluid layers and brine lake. For the subseafloor sediments, depth is given in cm below the top of the gravity core. Depth is given as CTD depth for the brine fluid, and as cable length for the fluid mud layer. Sodium and chloride concentration profiles are derived from Aiello et al. (2020). Presumable contaminants are shown with a white dot pattern.

Overlying the subseafloor sediments and fluidized muds of the Urania Basin like a lid, a massive brine layer extends between 3,463 and 3,610 mwd. The samples recovered in Niskin bottles returned clear brine fluids from the 3,465 m to 3,610 mwd interval (Figure 1). The redox potential in the brine lake was extremely negative at 3,560 mwd, i.e., –427 mV, consistent with previous results showing that oxygen was completely depleted in the reducing brine lake environment (Borin et al., 2009). The brine contained chloride and sodium at constant concentrations of 2,823 and 2,450 mM, respectively (Aiello et al., 2020), *ca*. 4.5 times higher than in the underlying fluid mud and subseafloor sediments (Figure 2).

For over 110 vertical meters below the brine layer, the Niskin bottles collected fluid mud (Figure 1) with much higher density (>1.6 g/cm^3^) than the brine (1.13 g/cm^3^), comparable to the bulk-saturated density of similar pelagic sediments (Fusi et al., 1996). The water content in the fluid mud ranges between ∼61 and 68%, which is above the Attemberg’s liquid limit measured for similar sediments recovered during ODP Leg 160 at the Olimpi Mud Volcano field (Kopf et al., 1998). The flat pore water profiles of Na^+^ and Cl^–^ across the fluid mud layer suggest some internal mixing to obscure internal biogeochemical stratification (Figure 2). Internal mixing is also supported by consistent total sulfur concentrations, dominated by sulfate, near 95 and 53 mM for brine and fluid mud, respectively (Aiello et al., 2020). Above-seawater salinities suggest that the fluid mud was mixed with pre-existing brine fluids. In lakes, temperature and salt differences between two bodies of water/sediment can lead to double-diffusive convection cells, which can highly enhance vertical transport of heat and salt (Boehrer, 2012). Consistent with the maximum depth reached by CTD rosette deployment in the fluid mud layers, the Gravity corer (GeoB15101-7) encountered a resistant sediment layer at about 3,735 m cable length. The Gravity corer returned 4.5 m of sediment, soupy and homogeneous within the upper 80 cm but increasingly firm below; the sediment at the very bottom of the core was consolidated.

### 3.2 Gases and hydrocarbon lipid biomarkers

Previously measured temperatures of 45°C in the bottom of the southwestern part of the basin (Corselli et al., 1996) are a strong indicator of a deep subsurface reservoir fueling fluids and mud to the Urania Basin seafloor. Since high pressure and temperature transform buried organic matter to hydrocarbons, the consolidated subseafloor sediment samples obtained by gravity coring were examined for methane and ethane, as well as hydrocarbon lipid biomarkers. The concentrations of methane and ethane are consistently high, 0.14–0.79 mM and 0.03–0.13 mM in the brine, respectively, and 0.4–3.3 mM and 0.07–0.60 mM, respectively, in the consolidated subseafloor sediments (Supplementary Table 1). The δ^13^C values of methane and ethane in the fluid mud layers are rather invariable (approximately –30.7 and –30.2‰, respectively) and nearly identical to δ^13^C values in the subseafloor sediment (approximately –30.3 and ∼ –29.1‰, respectively). Both are indicating a deep thermogenic source, which is corroborated by exceptionally low C_1_/C_2_ ratios of ∼5 in the fluid mud and in the sediments (Supplementary Table 1).

Polycyclic aromatic molecules in subseafloor sediments (Supplementary Table 2) included compounds typically found in petroleum such as naphthalene, fluorine, phenanthrene or pyrene but also high amounts of benzidine. The portion of polyaromatic hydrocarbons was high compared to the relative sum of identified hydrocarbons, including odd n-alkanes in the range from C_21_ to C_33_ as well as terpenoids such as squalene, Hop-17,21-ene and lycopane, i.e., 82.6% in the deep subseafloor sample (260–280 cm core depth) and 86.7% in the shallow sample (10–30 cm core depth). The extremely high proportion of thermally altered hydrocarbons detected in the subseafloor sediments of the Urania Basin is consistent with the hypothesis of a deep, hot reservoir that charges deep sediments and fluidized mud with gas of thermocatalytic or hydrothermal origin (Karisiddaiah, 2000; Hübner et al., 2003).

### 3.3 Microbial community of the Urania Basin brine

The bacterial and archaeal community of Urania basin, as evaluated by 16S rRNA gene sequencing (Table 1 and Supplementary Table 3), underwent pronounced compositional changes between brine, fluid mud and bottom sediment samples (Figure 2). Since these samples differ mainly by salinity, and less by biomarker content (only the bottom sediment) or by gas concentration and isotopic signature, microbial community differences are likely to be connected to salinity; extreme halophiles would be expected in the brine layer and might diminish in the fluid muds below.

**TABLE 1:** Summary of Urania Basin 16S rRNA gene sequences and their phylogenetic affiliations based on Genbank searches for Bacteria and Archaea.

Sample	Total clones	Proteobacteria	Other bacteria	Grampositives	Archaea
Brine layer/Sample 5	82 bact. clones 93 arch. clones	4 *Desulfatiglandales* (δ) 1 *D*. *natronobacter* (δ) 1 *Desulfococcus* sp. (δ) 1 *Desulfobulbales* (δ) 2 *Sulfurimonas* sp. (ε) 2 *Sulfurovum* sp. (ε) 4 *Arcobacter* sp. (ε)	10 MSBL5 1 Planctomycetales 2 Parcubacteria 3 MSBL2 1 MSBL2-related 1 Bacteroidetes 2 KB1 (type 1) 47 KB1 (type 2)		79 *Halodesulfoarchaeum* sp. 9 MSBL1 4 MSBL1 sister lineage 1 Thermoprofundales/MBGD
Upper fluid mud layer/Sample 8	59 bact. clones 90 arch. clones	6 *Stenotrophomonas* sp. (γ)* 3 *Cupriavidus* sp. (β)* 4 *Halomonas* sp. (γ)	4 *Thermus* sp.	7 *Bacillus* sp. 12 *Piscibacillus* sp. 3 *Anoxybacillus* sp. (near *A*. *flavithermus*) 12 *Anoxybacillus* sp. (near *A*. *geothermalis*) 7 *Geobacillus* sp. (*near G. pallidus*)	63 MBSL1 4 MSBL1 sister lineage 19 *Halodesulfoarchaeum* sp. 4 Bathyarchaeota/ MCG-17
Central fluid mud layer/Sample 14	57 bact. clones 95 arch. clones	1 *Bradyrhizobium* (α) 12 *Stenotrophomonas* sp.(γ)* 3 *Cupriavidus* sp. (β)* 4 *Halomonas* sp. (γ)	1 Chloroflexi 1 Spirochete	2 *Bacillus* sp. 1 *Terrilactibacillus* sp. 2 *Anoxybacillus* sp. (near *A*. f*lavithermu*s*)* 16 *Anoxybacillus* sp. (near *A. geothermalis*) 6 *Geobacillus* sp. (*near G. pallidus*) 1 *Geobacillus* sp. (near *G. stearothermophilus*) 7 uncult. Firmicutes	32 MSBL1 62 Thermoprofundales/MBGD 1 Marine Thermoplasmata
Lower fluid mud layer/Sample 21	56 bact. clones 93 arch. clones	4 *Bradyrhizobium* sp. (α) 6 *Stenotrophomonas* sp. (γ)* 1 *Cupriavidus* sp. (β)*	2 Acetothermia 1 *Thermus* sp.	12 *Anoxybacillus* sp. (near *A*. *flavithermus*) 24 *Anoxybacillus* sp. (near *A*. *geothermalis*) 5 *Geobacillus* sp. (near *G. pallidu*s) 1 *Caldialkalibacillus* sp.	2 MSBL1 1 *Halodesulfoarchaeum s*p. 90 Marine Thermoplasmata
Sediment core 10–30 cm	80 bact. clones 85 arch. clones	16 *Cycloclasticus* sp. (γ)		25 uncultured Firmicutes 39 *Veillonellaceae* sp.*	85 Urania Basin Euryarchaeotal Group (UBEG)
Sediment core 260–280 cm	75 bact. clones 73 arch. clones	12 *Stenotrophomonas* sp. (γ)* 18 *Delftia* sp. (β)* 7 *Acinetobacter* sp. (γ) 5 *Achromobacter* sp. (β)*		27 uncultured Firmicutes 6 *Propionibacterium* sp.*	38 Thermoprofundales/MBGD 20 Bathyarchaeota/MCG-8 10 Bathyarchaeota/ MCG-6 5 Lokiarchaeota

Sequence similarities in % to Genbank matches are listed in Supplementary Table 3. Asterisks identify likely contaminants.

**FIGURE 3:**
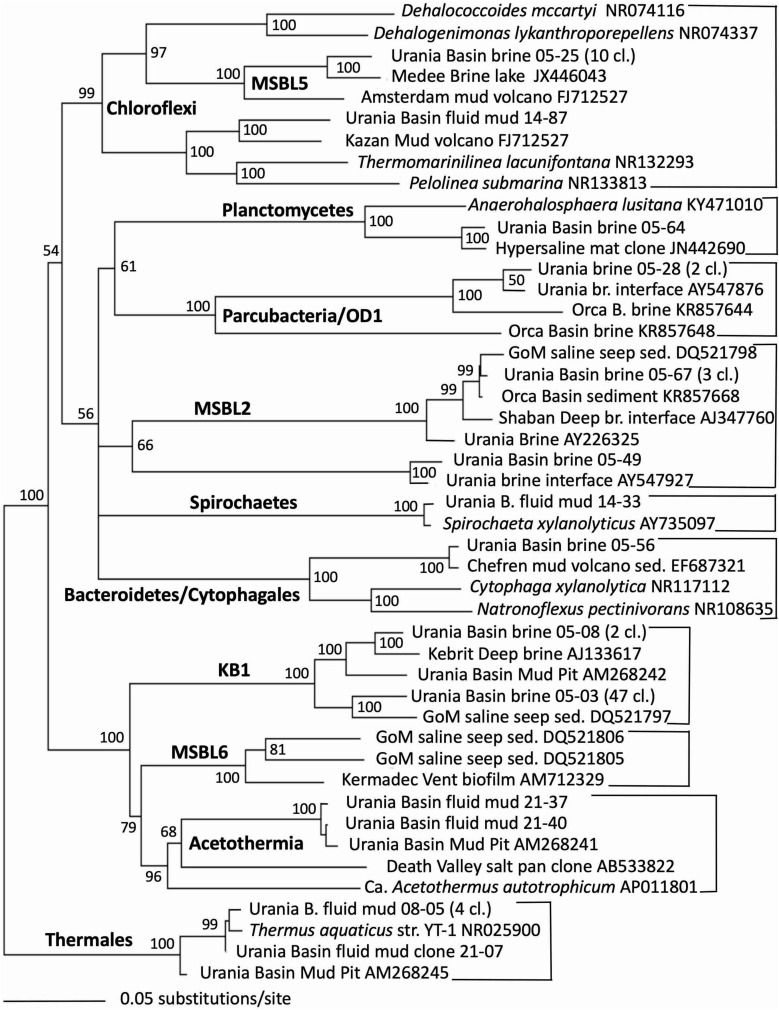
Phylogeny of general bacteria-affiliated 16S rRNA genes based on distance minimum evolution analysis and 1,000 neighbor-joining bootstrap iterations using PAUP (Swofford, 2000).

**FIGURE 4:**
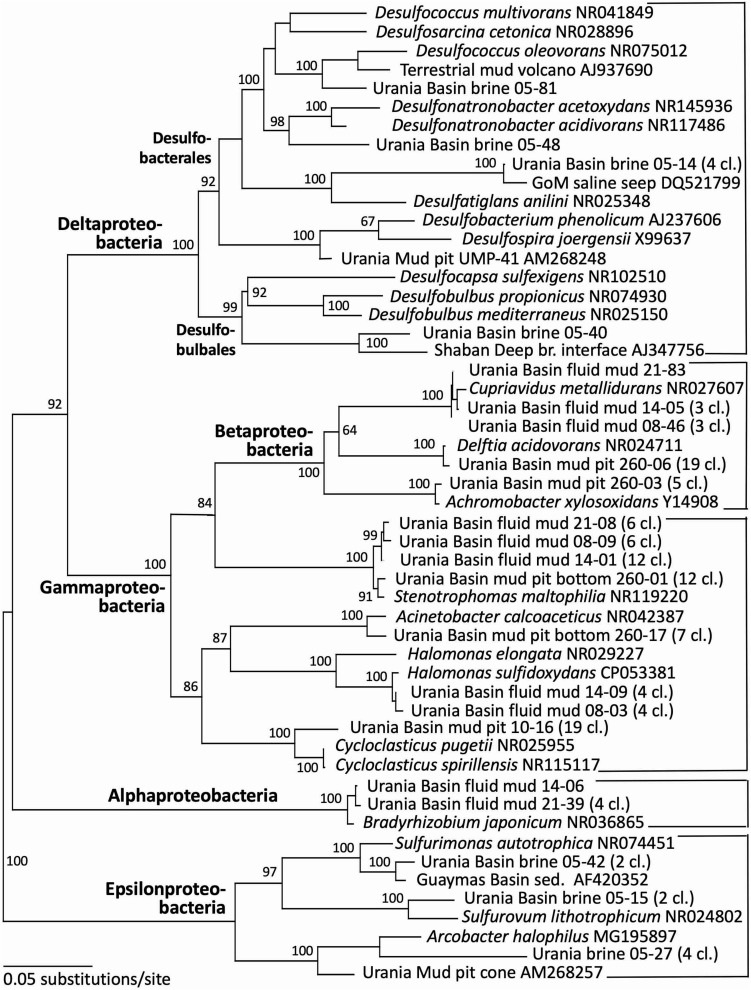
Phylogeny of the Proteobacteria-affiliated 16S rRNA genes based on distance minimum evolution analysis and 1,000 neighbor-joining bootstrap iterations using PAUP (Swofford, 2000).

Indeed, the uncultured halophilic Kebrit deep group 1 (KB-1) dominated the bacterial 16S rRNA data set from the brine sample (Figure 3 and Table 1). This bacterial lineage was first detected in brine sediments from the Kebrit Deep, Red Sea (Eder et al., 1999), and has so far been exclusively found in saline and hypersaline habitats, anoxic brines and sediments (Eder et al., 2001, 2002; Antunes et al., 2011; Ferrer et al., 2012). Yakimov et al. (2013) suggested that KB-1 could carry out reductive cleavage of glycine betaine and consequently produce acetate and methylamines, which could be used by halophilic methylotrophic H_2_-producing methanogens, such as the putatively methanogenic uncultured MSBL-1 group (Nigro et al., 2016). KB-1 also shares the carbon monoxide dehydrogenase (subunit CooS) and the Acetyl-CoA synthase (subunit AcsB) of the CO_2_-fixing acetyl-CoA pathway with *Candidatus* Acetothermum autotrophicum, a member of its sister lineage Acetothermia (Nigro et al., 2016), suggesting shared physiological flexibility with both autotrophic and acetogenic potential (Youssef et al., 2019). Six sequences were affiliated with sulfate-reducing bacteria (Figure 4). Three of these sequences were affiliated with the *Desulfobacterium anilini* group within the *Desulfobacteraceae*, a cluster of obligately aromatics-degrading sulfate-reducing bacteria that oxidize and remineralize substituted aromatics and polyaromatic substrates (Teske, 2019). Two sequences were related to the haloalkaliphilic genus *Desulfonatronobacter* (Sorokin et al., 2012), and one clone belonged to the family *Desulfobulbaceae*, a diverse group of incompletely oxidizing sulfate-reducing or elemental sulfur-disproportionating bacteria (Kuever et al., 2005). Generally, sulfate-reducing bacteria have access to high sulfate concentrations in the brine and fluid mud layers (Aiello et al., 2020). Eight sequences were affiliated with ε-Proteobacteria, chemolithoautotrophs that probably use the reverse TCA cycle to fix carbon, and usually thrive in sulfidic interface environments, for example the redoxcline of sulfidic waters (Schmidtova et al., 2009). Four sequences were affiliated to the genus *Arcobacter*, efficient CO_2_ fixers in hypoxic sulfide-rich habitats that oxidize sulfide to produce elemental sulfur while surviving under severe electron acceptor limitation (Wirsen et al., 2002). The remaining sequences were affiliated with the sulfide-oxidizing, nitrate-, or sulfur-reducing genera *Sulfurimonas* and *Sulfurovum* (Campbell et al., 2006). Sequences affiliated with uncultured, presumably halophilic bacterial lineages from seafloor brine lakes (Bannock Brine Basin) were recovered in the Urania brine, and include the MSBL5 lineage within the Chloroflexi, and the MSBL2 lineage that is currently not closely affiliated with other bacteria (Daffonchio et al., 2006). Other bacterial community members were represented by small numbers of clones of most likely heterotrophic and/or fermentative groups among the Planctomycetes, Parcubacteria (Castelle et al., 2017), and Bacteroidetes (Figure 3 and Table 1).

**FIGURE 5:**
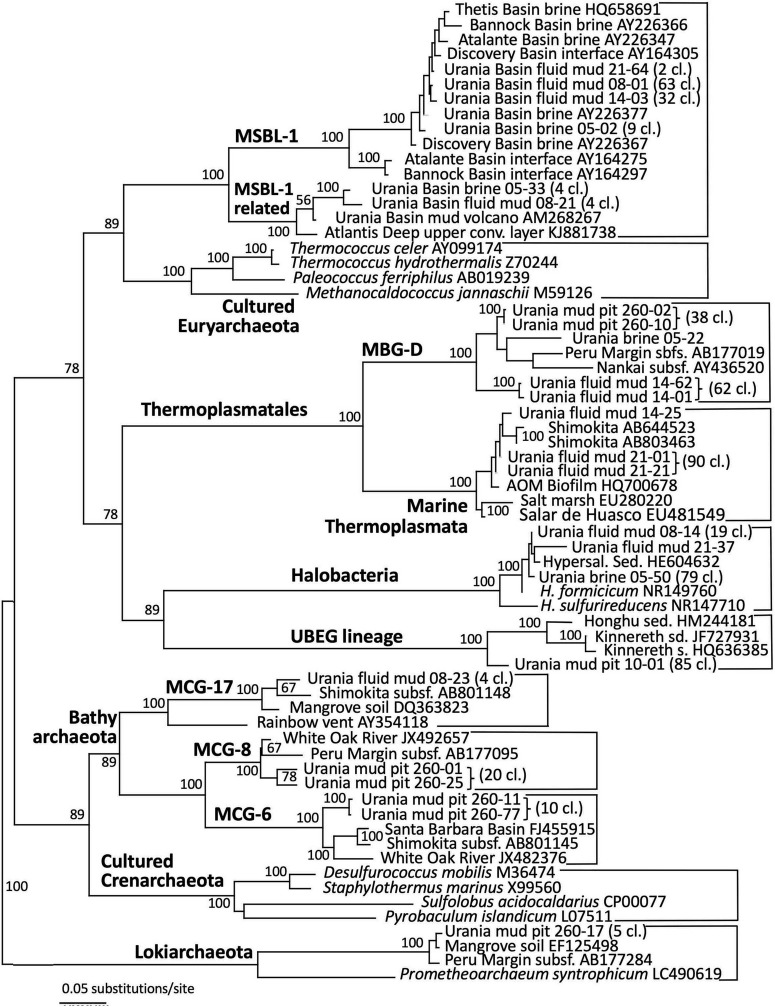
Phylogeny of the Archaea-affiliated 16S rRNA genes based on distance minimum evolution analysis and 1,000 neighbor-joining bootstrap iterations using PAUP (Swofford, 2000).

The archaeal sequences in the brine were dominated by anaerobic halophiles (Figure 5 and Table 1). Archaeal 16S rRNA gene sequences in the Urania Basin brine were closely related to *Halodesulfoarchaeum formicicum*, a lithoheterotroph that oxidizes formate or H_2_ with elemental sulfur, thiosulfate or DMSO as the electron acceptor, and to *Haloanaeroarchaeum sulfureducans*, which oxidizes acetate by sulfur respiration (Sorokin et al., 2018). The Urania Basin haloarchaeal counterparts to these cultured strict anaerobes are frequently recovered from the highly reducing and sulfide-rich Urania Basin brine. Other members of the archaeal brine community include members of the uncultured, presumably methanogenic MSBL1 lineage (Daffonchio et al., 2006) that grow potentially in syntrophy with KB1 bacteria (Yakimov et al., 2013), four clones of a MSBL1-related sister lineage, and a single clone of the heterotrophic Thermoprofundales (Zhou et al., 2019).

### 3.4 Microbial community of the Urania Basin fluid mud layers

**FIGURE 6:**
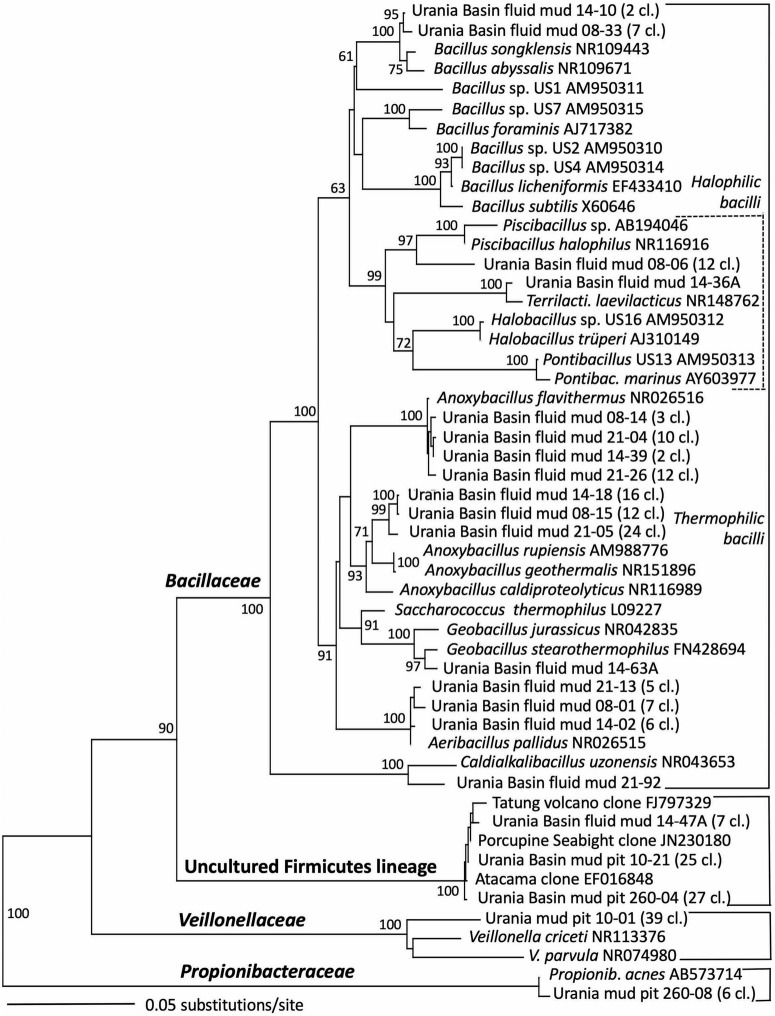
Phylogeny of the Firmicutes-affiliated 16S rRNA genes based on distance minimum evolution analysis and 1,000 neighbor-joining bootstrap iterations using PAUP (Swofford, 2000).

The taxonomic affiliation of bacterial sequences in the fluid mud layers changed considerably from those in the brine layer, to such a degree that the clone libraries showed no overlap at all (Figure 2 and Table 1). Instead of being affiliated with diverse sulfur-cycling delta- and epsilonproteobacteria and phylum-level halophilic lineages, most bacterial sequences of the three fluid mud layers comprised sequences specifically affiliated with genera of the family *Bacillaceae* within the Firmicutes (Figure 6). The upper fluid mud layer yielded a higher proportion of sequences affiliated with moderately halophilic and alkaliphilic *Bacillaceae* related to the genus *Piscibacillus* (Amoozegar et al., 2009), and—a little more distant but within the same monophyletic cluster—halophilic *Bacillaceae* of the genera *Terrilactibacillus*, *Halobacillus*, and *Pontibacillus* cultured from Urania Basin sediment (Sass et al., 2008). The halophile-related clones co-occur in the upper fluid mud layer with clones related to moderately thermophilic genera *Anoxybacillus* and *Geobacillus*, facultatively anaerobic chemoorganotrophs with a fermentative metabolism (Pikuta et al., 2000; Nazina et al., 2001). In the central and lower fluid mud layer, the halophile-related clones are no longer recovered, and the thermophilic lineages, especially *Anoxybacillus*, predominate. The *Anoxybacillus*-affiliated clones fall into two distinct clusters, one very similar to *Anoxybacillus flavithermus* (Pikuta et al., 2000), whereas the second and more frequently recovered cluster represents a sister lineage to the thermophilic, alkalitolerant, facultatively or strictly anaerobic species *A. rupiensis* and *A. geothermalis* (Filippidou et al., 2016). In contrast to the apparent halophile-thermophile transition, all fluid mud layers yielded almost equal numbers of clones affiliated with the thermophilic, alkalitolerant, but aerobic species *Geobacillus pallidus*, recently renamed *Aeribacillus pallidus* (Miñana-Galbis et al., 2010). We speculate that these populations represent a terrestrial import that remains detectable due to endospore formation.

Other *Bacillaceae*-affiliated lineages were recovered less frequently, or in only a single layer. Clones related to the marine species *Bacillus songklensis* (Kang et al., 2013) and *Bacillus abyssalis* (You et al., 2013) of the thermophilic, endospore-forming genus *Bacillus* were recovered from the upper and middle fluid mud horizon. A sequence each from the middle and bottom layer of the mud fluid were affiliated with the thermophilic, heterotrophic, alkalitolerant, and hydrocarbon-degrading species *Geobacillus stearothermophilus* (Nazina et al., 2001) and *Caldalkalibacillus uzonensis* (Zhao et al., 2008), respectively. Further, the central mud layer yielded clones of an uncultured Firmicutes lineage that has consistently close Genbank matches in diverse marine and terrestrial sediment and subsurface environments, and thus appears to represent an evolutionary lineage with a preference for these habitats (Figure 6 and Table 1).

Gamma-, Beta- and Alphaproteobacterial clones formed the second major bacterial group detected in the fluid mud layers, affiliated with the genera *Delftia*, *Cupriavidus*, *Stenotrophomonas*, *Halomonas* and *Bradyrhizobium* (Figure 4 and Table 1). Of these, the genus *Halomonas* (Vreeland et al., 1980) consisting of halotolerant or halophilic, aerobic or facultatively anaerobic isolates from diverse saline environments, occurs specifically in marine environments (Wang and Shao, 2021, and refs. therein). The *Halomonas* clones are closely related to *Halomonas sulfidoxydans*, a highly sulfide-tolerant marine sediment species that oxidizes sulfide aerobically or with nitrate or N_2_O as electron acceptor (Wang and Shao, 2021). *Rhizobium* strains were among the most consistently isolated bacteria from subsurface Mediterranean sapropels (Suess et al., 2004, 2006). In contrast, the hydrocarbon-degrading freshwater and soil bacterium and opportunistic pathogen *Delftia* (Wen et al., 1999; Højgaard et al., 2022), the heavy metal-resistant soil bacterium *Cupriavidus* (Vandamme and Coenye, 2004) and the plant-and human-associated bacterium *Stenotrophomonas* (Ryan et al., 2009) lack marine or subsurface associations. We caution that these sequences were most likely introduced as contaminants during handling of samples, reagents or PCRs, and we are reporting them to provide reference sequences for critical evaluations of microbial community surveys in extreme habitats (Table 1).

The upper and lower mud fluid samples yielded sequences related to *Thermus aquaticus*, a heterotrophic, thermophilic bacterium from thermal springs in Yellowstone National Park that uses several sugars and organic acids as carbon sources (Brock and Freeze, 1969). The genus contains aerobic as well as facultatively anaerobic strains, for example a facultatively anaerobic *Thermus* sp. isolated from South African Gold mines oxidizes organic compounds such as lactate with Fe (III) and/or Mn (IV) as electron acceptors (Kieft et al., 1999). Sequences affiliated with *Thermus* sp. were reported independently at 3,727 m depth in the deep fluid mud of the Urania Basin mud volcano (Yakimov et al., 2007), indicating that this thermophile is indeed a member of this microbial ecosystem.

The archaeal profile of the mud layers shows a transition from predominantly *Halodesulfoarchaeum*- and MSBL-1-affiliated clones in the top layer toward uncultured Thermoplasmatales lineages, specifically the Thermoprofundales [formerly Marine Benthic Group D (MBG-D)] in the middle layer and a previously undescribed Thermoplasmata lineage in the bottom layer (Figure 4 and Table 1). Extensive Genbank searches and comparisons with published phylogenies specified the phylogenetic position of the Urania Basin clones within the globally distributed and highly diverse Thermoplasmatales. The clones from the middle mud layer were affiliated with the marine sediment subclusters 6, 7, and 8 of the Thermoprofundales (Zhou et al., 2019), indicating tolerance to marine salinity at a minimum. MBG-D sequences have previously been found in abundance in hypersaline, sulfidic methane seep sediments in the Gulf of Mexico (Lloyd et al., 2006), and MBG-D was the major archaeal group in highly sulfidic anoxic sediments of the Salton Sea (Swan et al., 2010). The marine Thermoplasmata clones of the lower mud layer represented a sister lineage of the uncultured DHVE-9/20c-4 clade (Durbin and Teske, 2012) that was originally found at hydrothermal vents (Pagé et al., 2004). Genomic reconstruction has predicted that marine subsurface Thermoplasmatales are involved in detrital matter degradation, as well as acetogenesis (Lloyd et al., 2013; Lazar et al., 2017). The Urania Basin phylotypes were only distantly related to cultured sulfur-and iron-reducing Thermoplasmatales, for example the genera *Thermoplasma*, *Picrophilus*, or *Aciduliprofundum*.

Detection of MSBL1 archaea-related sequences is consistent with previous observations of this group in Urania Basin mud fluids (Yakimov et al., 2007) and the brine lake of the Urania Basin (van der Wielen et al., 2005). The MSBL1 archaea have been detected in many hypersaline environments, such as an endoevaporitic microbial mat (Sørensen et al., 2005), the Bannock, Urania and Thetis brine lakes (Daffonchio et al., 2006; Borin et al., 2009; La Cono et al., 2011), or Tunisian multipond solar salterns (Baati et al., 2008).

### 3.5 Microbial community of the Urania Basin subseafloor sediments

Sequences from the subseafloor sediments (the “mud pit”) differed considerably from those in the overlying fluid mud layers and brine (Figure 2). The uncultured subsurface Firmicutes lineage-affiliated clones that were previously identified in the middle fluid mud layer occurred in larger proportions in both subseafloor sediment samples (Figure 6 and Table 1). The upper gravity core sediments (10–30 cm) yielded sequences affiliated with the fermentative, anaerobic genus *Veillonella*, a widely occurring inhabitant of animal and human intestinal and oral mucosa and an unlikely community member of Urania Basin (van den Bogert et al., 2013). The lower core section (260–280 cmbsf) yielded sequences closely related to the fermentative Actinobacterium *Propionibacterium acnes*, reclassified as *Cutibacterium acnes*, a common epibiont on human skin (Scholz and Kilian, 2016). Due to their likely human origin, we regard the *Veillonella* and *Cutibacterium* sequences as contaminants.

Gammaproteobacterial sequences recovered from the upper gravity core sediments (10–30 cmbsf) were affiliated with the genus *Cycloclasticus*, which utilizes various aromatic hydrocarbons such as naphthalene, phenanthrene, biphenyltoluene, xylene or pyrene as the sole carbon and energy source (Wang et al., 2008; Lai et al., 2012). Cultured representatives of the genus *Cycloclasticus* are strictly aerobic, but their counterparts in anaerobic Urania Basin sediments might represent a related but distinct anaerobic group, compatible with the sequence divergence (*ca*. 4%) between the cultured strains the Urania Basin clones (Figure 4). Clones from the deeper core section (260–280 cmbsf) were affiliated with the gammaproteobacterial genus *Acinetobacter*, capable of hydrocarbon degradation in hydrocarbon-polluted soils or in oilfield production liquids (Chaîneau et al., 1995; Gong et al., 2012). Sequences of the gamma- and betaproteobacterial genera *Stenotrophomonas* (associated with human clinical samples; Ryan et al., 2009), *Delftia* (an opportunistic emergent pathogen; Højgaard et al., 2022) and *Achromobacter* (described from human sample material; Yabuuchi and Yano, 1981), were found in the deep gravity core sample (260–280 cmbsf) and represent likely contaminants from human contact and handling in the lab.

Archaeal sequence datasets from the upper layers of the gravity core (10–30 cmbsf) were exclusively composed of an uncultured Euryarchaeotal Group (Figure 5). This lineage is not phylogenetically close to any known cultured or uncultured archaeal lineage, and the closest uncultured clones were retrieved from lake sediments or Arctic thermal springs. Extensive chimera checks of whole and partial sequences did not uncover any evidence for a mosaic sequence; all sections of the 16S rRNA gene remained ≈15% different from other sequences in Genbank. We called this group Urania Basin Euryarchaeota Group (UBEG).

The deeper gravity core sample (260–280 cmbsf) was dominated by the same uncultured Thermoprofundales lineage as in the middle fluid mud layer, and by heterotrophic, anaerobic and presently uncultured Bathyarchaeota of the MCG-6 and MCG-8 subgroups (Kubo et al., 2012). These Bathyarchaeota were distinct from the subgroup MCG-17 clones that were recovered in the upper fluid mud layer (Figure 5). Of these subgroups, MCG-6 has a wide environmental distribution in soil, hot springs, estuarine and marine sediments, whereas MCG-8 and MCG-17 are specifically associated with estuarine and marine sediments (Xiang et al., 2016). Finally, sequences affiliated with the Lokiarchaeota (Spang et al., 2015) were found in smaller numbers. Mixed archaeal communities of Thermoplasmatales, Bathyarchaeota and Lokiarchaeota are characteristic for marine sediments, and were already found in the first major sequencing survey of marine deep-sea sediments (Vetriani et al., 1999).

## 4 Discussion

The bacterial and archaeal communities of the Urania Basin brine fluids, fluid mud layers and deep sediments show characteristic, habitat-related trends as they change throughout the sample series, after discounting contaminant sequences affiliated to bacteria associated with medical, human, or animal samples (*Achromobacter*, *Delfia*, *Propionibacterium, Stenotrophomonas*, *Veillonellaceae*) or terrestrial soil (*Cupriavidus*). Sequences affiliated with sulfur-cycling Epsilon- and Deltaproteobacteria, extremely halophilic KB1 bacteria, and extremely halophilic *Halodesulfoarchaeum* spp. in the brine are replaced by sequences affiliated with diverse Firmicutes, and by halophilic archaea (putatively methanogenic MSBL-1) and Thermoplasmatales in the fluid mud layers. Toward deeper fluid mud layers the Firmicutes change from halophilic (*Piscibacillus*) to thermophilic (*Anoxybacillus*) lineages, and the Archaea change from extreme halophiles (*Halodesulfoarchaeum* and MSBL1) toward subsurface sediment lineages (Thermoprofundales and Thermoplasmata). Finally, the deep subsurface sediment below the mud volcano retains sequences related to uncultured Firmicutes, hydrocarbon-degrading Gammaproteobacteria (*Cycloclasticus* spp., *Acinetobacter* spp.), and marine subsurface archaea of the Bathyarchaeota, Thermoprofundales, and Lokiarchaeota, plus an unidentified archaeal group that appears only in the upper gravity core sample. Nearly all bacterial and archaeal clones are affiliated to heterotrophic groups, indicating that this downward microbial succession (brine—fluid mud—subsurface sediment) degrades organic substrates and hydrocarbons that are available in the brine, fluid mud layers, and subsurface sediment.

The mud volcano fluids and subsurface sediment microbial communities resemble each other in the presence of gram-positive, sometimes thermophilic, and presumably spore-forming genera and families within the phylum Firmicutes. Thus, the Urania Basin sediment microbiota provide an illustrative example for a “firmicute hotspot,” the previously postulated point sources that distribute endospore-forming, moderately thermophilic Firmicutes across cold marine sediments world-wide (Hubert et al., 2009; Müller et al., 2014; Gittins et al., 2022). These spore-forming bacteria require a certain minimum temperature but cannot grow in deep-sea surficial sediments that are permanently cold. Possible sources include hydrothermal vents, mud volcanoes, and terrestrial sedimentation. Hydrothermal vents have generally very low proportions of gram-positive bacteria, which require lab enrichment for detection (Castro et al., 2021). Terrestrial imports appear likely in some coastal locations (Lee et al., 2005). However, mud volcanoes are the most promising source of these widespread seafloor thermophiles (Rattray et al., 2022). The Urania Basin Mud volcano, prone to occasional eruptions (Hübner et al., 2003) adds to the database of sources for widely spread gram-positive bacteria in the deep-sea. We speculate further that the carbon and energy source for at least some of these Firmicutes might be petroleum-derived hydrocarbons of deep subsurface origin, since polyaromatic compounds and long-chain alkanes are abundant in the fluidized mud and subseafloor sediment of Urania Basin.

The high contribution of gram-positive Firmicutes in this study stands in contrast to the near-absence of gram-positive bacteria (a single Actinomycete clone) in a previous survey of the Urania mud volcano (Yakimov et al., 2007). These differences might reflect different nucleic acid extraction protocols and PCR targets: chemical extraction of RNA *via* QIAGEN RNA/DNA mini extraction kit followed by reverse transcription to cDNA (Yakimov et al., 2007) vs. bead-beating cell breakage before kit-based DNA extraction and column purification in this study. The mechanical force of bead-beating has very likely increased the recovery of DNA from spore-forming gram-positive bacteria. When bead-beating techniques are used for cell disruption and DNA extraction, gram-positive spore-forming bacteria are recovered from diverse marine sediments (for example, Teske et al., 2002). We note that any DNA-based study might include dead or dormant cells. We also note that the previous study did not involve a nested PCR approach (Yakimov et al., 2007), and thus avoided a likely source of contaminating sequences.

Active sulfur and methane cycling characterize the brine/seawater transition of Urania Basin, where seawater sulfate and subsurface methane overlap (Borin et al., 2009). At present, our data do not rule out active sulfur and methane cycling in the deep brine and the mud volcano fluid. Although the PCR data of this study indicate that sequences affiliated with microbial taxa involved in sulfur cycling appear to be limited to the brine layer, sequences related to sulfur-oxidizing Epsilon- and sulfate-reducing Deltaproteobacteria have been recovered as dominant and active groups by rRNA transcript sequencing from deep fluid mud of Urania Basin (Yakimov et al., 2007). The mud volcano sediments and the overlying brine contain millimolar amounts of methane and sulfate (Borin et al., 2009; Zabel, 2012) and would in principle allow sulfate-dependent methane oxidation to take place. Although anaerobic sulfate-dependent methane-oxidizing archaea (ANME) were not found in this survey, they were detected as a single clone in the deep mud volcano fluid (Yakimov et al., 2007). Few studies have reported on ANME archaea in hypersaline environments. ANME-1 archaea were detected in a moderately briny, sulfidic methane seep in the Gulf of Mexico (Lloyd et al., 2006), and (in low numbers) in the bottom sediments of Orca Basin in the Gulf of Mexico (Nigro et al., 2020). ANME1 and ANME2 archaea were found in the Red Sea seafloor brine lakes (Atlantis II and Discovery Deep), predominantly in a sediment sample with high total sulfur content (Siam et al., 2012). A consensus reading of the available data indicates that small populations of ANME archaea exist in hypersaline basins and in the Urania Basin mud volcano, but these hard-to-find sequences are not comparable to the global microbial community signature of marine methane seeps, methane/sulfate interfaces or methane-rich subsurface sediments that are dominated by ANME archaea and deltaproteobacterial sulfate reducers (Ruff et al., 2015). Accordingly, we do not observe any evidence of decreasing concentrations of ^13^C-isotope enrichments in the methane pool.

Overall, this study highlights the diversity of bacteria and archaea thriving in the extremely harsh conditions of the Urania Basin brine lake and mud volcano. Further activity measurement or culture-based experiments could help understand which microbial metabolisms play a role in the Urania Basin, or how the microbes work together to survive in such an environment.

## Data availability statement

The datasets presented in this study can be found in online repositories. The names of the repository/repositories and accession number(s) can be found in the article/Supplementary material.

## Author contributions

CL collected Urania Basin samples, extracted DNA, performed PCR amplification, cloned the PCR amplicons, and submitted them for sequencing, performed the first set of phylogenetic analyses, and wrote the first version of this manuscript. FS, ME, and VH performed and tabulated geochemical analyses of Urania Basin samples, including the methane and ethane analyses. K-UH obtained funding for the DARC LIFE project and for the Expedition to Urania Basin and developed the scientific rationale for sampling site selection. AT advised CL on the sequencing project, prepared the phylogenetic trees in this manuscript, and wrote the second version of the manuscript. All authors revised and finalized the manuscript.

## Footnotes

1
http://www.arb-silva.de/aligner

## Supplementary material

Supplementary Table 1 Concentration and ^13^C stable isotope values of methane and ethane in Urania Basin brine, fluid mud, and subsurface sediment samples.

Supplementary Table 2 Concentrations of hydrocarbons in the subseafloor of the Urania Basin. PAH/HC, portion of polyaromatic hydrocarbons relative to sum of all identified hydrocarbons; Thermal HC/HC, portion of thermally altered hydrocarbons relative to sum of all identified hydrocarbons.

Supplementary Table 3 Similarities of Urania Basin phylotypes and related sequences of pure cultures and environmental phylotypes. Genbank numbers for bacteria and archaea are: OP389385-OP389468 and OP389469-OP389561 in sample 5; OP352680-OP352749 and OP389299-OP389384 in sample 8; OP352780-OP389844 and OP389717-OP389809 in sample 14; OP389562-OP389631 and OP389897-OP389989 in sample 21; OP352539-OP352606, OP389632-OP389716, and OP856603-OP856618 in sample GC-10-30; OP352609-OP352677, OP389811-OP389896, and OP856619-OP856629 for sample GC-260-280.

Supplementary Data Sheet 1 Supplementary methods.

## References

[B1] Aiello I. W. Beaufort L. Goldhammer T. Heuer V. B. Hinrichs K.-U. Zabel M. (2020). Anatomy of a ‘suspended’ seafloor in the dense brine waters of the deep hypersaline Urania basin. *Deep Sea Res. II* 171:104626. 10.1016/j.dsr2.2019.07.014

[B2] Aloisi G. Cita M. B. Castradori D. (2006). Sediment injection in the pit of the Urania anoxic brine lake (Eastern Mediterranean). *Rend. Lincei Sci. Fis.* 17 243–262. 10.1007/BF02904765

[B3] Amoozegar M. A. Sánchez-Porro C. Rohban R. Hajighasemi M. Ventosa A. (2009). *Piscibacillus halophilus* sp. nov., a moderately halophilic bacterium from a hypersaline Iranian lake. *Int. J. Syst. Evol. Microbiol.* 59 3095–3099. 10.1099/ijs.0.012013-0 19643892

[B4] Antunes A. Ngugi D. K. Stingl U. (2011). Microbiology of the Red Sea (and other) deep-sea anoxic brine lakes. *Environ. Microbiol. Rep.* 3 416–433. 10.1111/j.1758-2229.2011.00264.x 23761304

[B5] Baati H. Guermazi S. Amdouni R. Gharsalla N. Sghir A. Ammar E. (2008). Prokaryotic diversity of a Tunisian multipond solar saltern. *Extremophiles* 12 505–518. 10.1007/s00792-008-0154-x 18373061

[B6] Boehrer B. (2012). “Double-diffusive convection in lakes,” in *Encyclopedia of lakes and reservoirs. Encyclopedia of earth sciences series*, eds Bengtsson L. Herschy R. W. Fairbridge R. W. (Dordrecht, NL: Springer).

[B7] Borin S. Brusetti L. F. Mapelli G. D’Auria T. Brusa M. Marzorati A. (2009). Sulfur cycling and methanogenesis primarily drive microbial colonization of the highly sulfidic Urania deep hypersaline basin. *Proc. Natl. Acad. Sci. U.S.A.* 106 9151–9156. 10.1073/pnas.0811984106 19470485 PMC2685740

[B8] Brock T. D. Freeze H. (1969). *Thermus aquaticus* gen. n. and sp. n., a nonsporulating extreme Thermophile. *J. Bacteriol.* 98 289–297. 10.1128/jb.98.1.289-297.1969 5781580 PMC249935

[B9] Campbell B. J. Engel A. S. Porter M. L. Takai K. (2006). The versatile epsilon-proteobacteria: Key players in sulphidic habitats. *Nat. Rev. Microbiol.* 6 458–468. 10.1038/nrmicro1414 16652138

[B10] Casamayor E. O. Schäfer H. Baneras L. Salio C. P. Muyzer G. (2000). Identification of and spatio-temporal differences between microbial assemblages from two neighboring sulfurous lakes: Comparison by microscopy and denaturing gradient gel electrophoresis. *Appl. Environ. Microbiol.* 66 499–508. 10.1128/AEM.66.2.499-508.2000 10653710 PMC91855

[B11] Castelle C. J. Brown C. T. Thomas B. C. Williams K. H. Banfield J. F. (2017). Unusual respiratory capacity and nitrogen metabolism in a *Parcubacterium* (OD1) of the candidate phyla radiation. *Sci. Rep.* 7:40101. 10.1038/srep40101 28067254 PMC5220378

[B12] Castro S. P. Borton M. A. Regan K. Hrabe de Angelis I. Wrighton K. C. Teske A. P. (2021). Degradation of biological macromolecules supports uncultured microbial populations in Guaymas Basin hydrothermal sediments. *ISME J.* 15 3480–3497. 10.1038/s41396-021-01026-5 34112968 PMC8630151

[B13] Chaîneau C.-H. Morel J.-L. Oudot J. (1995). Microbial degradation in soil microcosms of fuel oil hydrocarbons from drilling cuttings. *Environ. Sci. Technol.* 29 1615–1621. 10.1021/es00006a027 22276886

[B14] Charlou J. L. Donval J. P. Zitter T. Roy N. Jean-Baptiste P. Foucher J. P. (2003). Evidence of methane venting and geochemistry of brines on mud volcanoes of the Eastern Mediterranean Sea. *Deep Sea Res.* 50 941–958. 10.1016/S0967-0637(03)00093-1

[B15] Corselli C. Basso D. de Lange G. Thomson J. (1996). Mediterranean ridge accretionary complex yields rich surprises. *EOS* 77 227–227. 10.1029/96EO00159

[B16] D’Hondt S. L. Jørgensen B. B. Miller D. J. (2003). *Proc. ODP, Init. Repts., 201.* College Station, TX: Ocean Drilling Program.

[B17] Daffonchio D. Borin S. Brusa T. Brusetti L. van der Wielen P. W. Bolhuis H. (2006). Stratified prokaryote network in the oxic–anoxic transition of a deep-sea halocline. *Nature* 440 203–207. 10.1038/nature04418 16525471

[B18] Durbin A. M. Teske A. (2012). Archaea in organic-lean and organic-rich marine subsurface sediments: An environmental gradient reflected in distinct phylogenetic lineages. *Front. Microbiol.* 3:168. 10.3389/fmicb.2012.00168 22666218 PMC3364523

[B19] Eder W. Jahnke L. L. Huber R. (2001). Microbial diversity of the brine-seawater interface of the Kebrit deep, Red Sea, studied via 16S rRNA gene sequences and cultivation methods. *Appl. Environ. Microbiol.* 67 3077–3085. 10.1128/AEM.67.7.3077-3085.2001 11425725 PMC92984

[B20] Eder W. Ludwig W. Huber R. (1999). Novel 16S rRNA gene sequences retrieved from highly saline brine sediments of Kebrit deep, Red Sea. *Arch. Microbiol.* 172 213–218. 10.1007/s002030050762 10525737

[B21] Eder W. Schmidt M. Koch M. Garbe-Schönberg D. Huber R. (2002). Prokaryotic phylogenetic diversity and corresponding geochemical data of the brine–seawater interface of the Shaban deep, Red Sea. *Environ. Microbiol.* 4 758–763. 10.1046/j.1462-2920.2002.00351.x 12460284

[B22] Ferrer M. Werner J. Chernikova T. N. Bargiela R. Fernández L. La Cono V. (2012). Unveiling microbial life in the new deep-sea hypersaline Lake Thetis. Part II: A metagenomic study. *Environ. Microbiol.* 14 268–281. 10.1111/j.1462-2920.2011.02634.x 22040283

[B23] Filippidou S. Jaussi M. Junier T. Wunderlin T. Jeanneret N. Plamieri F. (2016). *Anoxybacillus geothermalis* sp. nov., a facultatively anaerobic, endospore-forming bacterium isolated from mineral deposits in a geothermal station. *Int. J. Syst. Evol. Microbiol.* 66 2944–2951. 10.1099/ijsem.0.001125 27126386

[B24] Fusi N. Aloisi de Larderel G. Borello A. Amelio O. Castradori D. Negri A. (1996). Marine geology of the MEDRIFF corridor, Mediterranean Ridge. *Isl. Arc* 5 420–439. 10.1111/j.1440-1738.1996.tb00163.x

[B25] Gittins D. A. Desiage P.-A. Morrison N. Rattray J. E. Bhatnagar S. Chakraborty A. (2022). Geological processes mediate a microbial dispersal loop in the deep biosphere. *Sci. Adv.* 8:eabn3485. 10.1126/sciadv.abn3485 36026445 PMC9417182

[B26] Gong X. C. Liu Z. S. Guo P. Chi C. Q. Chen J. Wang X. B. (2012). Bacteria in crude oil survived autoclaving and stimulated differentially by exogenous bacteria. *PLoS One* 7:e40842. 10.1371/journal.pone.0040842 23028421 PMC3444520

[B27] Hall T. A. (1999). BioEdit: A user-friendly biological sequence alignment editor and analysis program for Windows 95/98/NT. *Nucl. Acids Symp. Ser.* 41 95–98.

[B28] Heuer H. Krsek M. Baker P. Smalla K. Wellington E. M. (1997). Analysis of actinomycetes communities by specific amplification of genes encoding 16S rRNA and gel-electroophoretic separation in denaturating gradients. *Appl. Environ. Microbiol.* 63 3233–3241. 10.1128/aem.63.8.3233-3241.1997 9251210 PMC168621

[B29] Højgaard S. M. M. Rezahosseini O. Knudsen J. D. Fuglebjerg N. J. U. M. Skov M. Nielsen S. D. (2022). Characteristics and outcomes of patients with *Delftia acidovorans* infections: A retrospective cohort study. *Microbiol. Spectr.* 10:e0032622. 10.1128/spectrum.00326-22 35862984 PMC9431703

[B30] Hoshino T. Toki T. Ijiri A. Morono Y. Machiyama H. Ashi J. (2017). Atribacteria from the subseafloor sedimentary biosphere disperse to the hydrosphere through submarine mud volcanoes. *Front. Microbiol.* 8:1135. 10.3389/fmicb.2017.01135 28676800 PMC5476839

[B31] Hubert C. Loy A. Nickel M. Arnosti C. Baranyi C. Brüchert V. (2009). A constant flux of diverse thermophilic bacteria into the cold arctic seabed. *Science* 325 1541–1544. 10.1126/science.1174012 19762643

[B32] Hübner A. De Lange G. J. Dittmer J. Halbach P. (2003). Geochemistry of an exotic sediment layer above sapropel S-1: Mud expulsion from the Urania Basin, Eastern Mediterranean? *Mar. Geol.* 197 49–61. 10.1016/S0025-3227(03)00085-9

[B33] Joye S. B. Samarkin V. A. Orcutt B. N. MacDonald I. R. Hinrichs K.-U. Elvert M. (2009). Metabolic variability in seafloor brines revealed by carbon and sulphur dynamics. *Nat. Geosci.* 2 349–354. 10.1038/ngeo475

[B34] Kang H. Weerawongwiwat V. Kim J.-H. Sukhoom A. Kim W. (2013). *Bacillus songklensis* sp. nov., isolated from soil. *Int. J. Syst. Evol. Microbiol.* 63 4189–4195. 10.1099/ijs.0.050682-0 23771626

[B35] Karisiddaiah S. M. (2000). Diverse methane concentrations in anoxic brines and underlying sediments, eastern Mediterranean Sea. *Deep Sea Res. I* 47 1999–2008. 10.1016/S0967-0637(00)00010-8

[B36] Kieft T. L. Fredrickson J. K. Onstott T. C. Gorby Y. A. Kostandarithes H. M. Bailey T. J. (1999). Dissimilatory reduction of Fe(III) and other electron acceptors by a *Thermus* isolate. *Appl. Environ. Microbiol.* 65 1214–1221. 10.1128/AEM.65.3.1214-1221.1999 10049886 PMC91167

[B37] Kopf A. Robertson A. H. F. Clennell M. B. Flecker R. (1998). Mechanism of mud extrusion on the Mediterranean Ridge accretionary complex. *Geo Mar. Lett.* 18 97–114. 10.1007/s003670050058

[B38] Kubo K. Lloyd K. G. Biddle J. F. Amann R. Teske A. Knittel K. (2012). Archaea of the miscellaneous crenarchaeotal group (MCG) are abundant, diverse and widespread in marine sediments. *ISME J.* 6 1949–1965. 10.1038/ismej.2012.37 22551871 PMC3449235

[B39] Kuever J. Rainey F. A. Widdel F. (2005). “Order III. Desulfobacterales ord. nov.: Family II. Desulfobulbaceae ord. nov. fam. nov,” in *Bergey’s manual of systematic bacteriology*, eds Garrity G. Brenner D. Krieg N. (Berlin: Springer), 988–998.

[B40] La Cono V. Smedile F. Bortoluzzi G. Arcadi E. Maimone G. Messina E. (2011). Unveiling microbial life in new deep-sea hypersaline Lake Thetis. Part I: Prokaryotes and environmental settings. *Environ. Microbiol.* 13 2250–2268. 10.1111/j.1462-2920.2011.02478.x 21518212

[B41] Lai Q. Li W. Wang B. Yu Z. Shao Z. (2012). Complete genome sequence of the pyrene-degrading bacterium *Cycloclasticus* sp. strain P1. *J. Bacteriol.* 194:6677. 10.1128/JB.01837-12 23144416 PMC3497472

[B42] Langille M. Zaneveld J. Caporaso J. MacDonald D. Knights D. Reyes J. A. (2013). Predictive functional profiling of microbial communities using 16S rRNA marker gene sequences. *Nat. Biotechnol.* 31 814–821. 10.1038/nbt.2676 23975157 PMC3819121

[B43] Lazar C. S. Baker B. J. Seitz K. Hinrichs K.-U. Teske A. (2017). Genomic reconstruction of multiple lineages of uncultured benthic archaea suggests distinct biogeochemical roles and ecological niches. *ISME J.* 11 1118–1129. 10.1038/ismej.2016.189 28085154 PMC5398341

[B44] Lazar C. S. L’Haridon S. Pignet P. Toffin L. (2011a). Archaeal populations in hypersaline sediments underlying orange microbial mats in the Napoli mud volcano. *Appl. Environ. Microbiol.* 77 3120–3131. 10.1128/AEM.01296-10 21335391 PMC3126394

[B45] Lazar C. S. Parkes R. J. Cragg B. A. L’Haridon S. Toffin L. (2011b). Methanogenic diversity and activity un hypersaline sediments of the centre of the Napoli mud volcano, Eastern Mediterranean Sea. *Environ. Microbiol.* 13 2078–2091. 10.1111/j.1462-2920.2011.02425.x 21382146

[B46] Lazar C. S. Parkes R. J. Cragg B. A. L’Haridon S. Toffin L. (2012). Methanogenic activity and diversity in the centre of the Amsterdam mud volcano, Eastern Mediterranean Sea. *FEMS Microbiol. Ecol.* 81 1–12. 10.1111/j.1574-6941.2012.01375.x 22458514

[B47] Lee Y.-J. Wagner I. D. Brice M. E. Kevbrin V. V. Mills G. L. Romanek C. S. (2005). Thermosediminibacter oceani gen. nov., sp. nov., and Thermosediminibacter litoperuensis sp. nov., new anaerobic thermophilic bacteria isolated from Peru Margin. *Extremophiles* 9 375–383. 10.1007/s00792-005-0453-4 15965715

[B48] Lloyd K. G. Lapham L. Teske A. (2006). An anaerobic methane-oxidizing community of ANME-1 archaea in hypersaline Gulf of Mexico sediments. *Appl. Environ. Microbiol.* 72 7218–7230. 10.1128/AEM.00886-06 16980428 PMC1636178

[B49] Lloyd K. G. Schreiber L. Petersen D. G. Kjeldsen K. U. Lever M. A. Steen A. D. (2013). Predominant archaea in marine sediments degrade detrital proteins. *Nature* 496 215–218. 10.1038/nature12033 23535597

[B50] Ludwig W. Strunk O. Westram R. Richter L. Meier H. Buchner A. (2004). ARB: A software environment for sequence data. *Nucleic Acids Res.* 32 1363–1371. 10.1093/nar/gkh293 14985472 PMC390282

[B51] McDonald I. R. Buthman D. B. Sager W. W. Peccini M. B. Guinasso N. L. (2000). Pulsed oil discharge from a mud volcano. *Geology* 28 907–910. 10.1130/0091-7613(2000)28<907:PODFAM>2.0.CO;2

[B52] Miñana-Galbis D. Pinzón D. L. Lorén J. G. Manresa Á Oliart-Ros R. M. (2010). Reclassification of *Geobacillus pallidus* (Scholz et al 1988) Banat et al 2004 as *Aeribacillus pallidus* gen. nov., comb. nov. *Int. J. Syst. Evol. Microbiol.* 60 1600–1604. 10.1099/ijs.0.003699-0 19700455

[B53] Müller A. L. de Rezende J. R. Hubert C. R. J. Kjeldsen K. U. Lagkouvardos I. Berry D. (2014). Endospores of thermophilic bacteria as tracers of microbial dispersal by ocean currents. *ISME J.* 8 1153–1165. 10.1038/ismej.2013.225 24351936 PMC4030223

[B54] Nazina T. N. Tourova T. P. Poltaraus A. B. Novikova E. V. Grigoryan A. A. Ivanova A. E. (2001). Taxonomic study of aerobic thermophilic bacilli: Descriptions of *Geobacillus subterraneus* gen. nov., sp. nov. and *Geobacillus uzenensis* sp. nov. from petroleum reservoirs and transfer of *Bacillus stearothermophilus*, *Bacillus thermocatenulatus*, *Bacillus thermoleovorans*, *Bacillus kaustophilus*, *Bacillus thermodenitrificans* to *Geobacillus* as the new combinations *G. stearothermophilus*. *Int. J. Syst. Evol. Microbiol.* 51 433–446. 10.1099/00207713-51-2-433 11321089

[B55] Nigro L. M. Elling F. J. Hinrinchs K.-U. Joye S. B. Teske A. (2020). Microbial ecology and biogeochemistry of hypersaline sediments in Orca Basin. *PLoS One* 15:e0231676. 10.1371/journal.pone.0231676 32315331 PMC7173876

[B56] Nigro L. M. Hyde A. S. MacGregor B. J. Teske A. (2016). Phylogeography, salinity adaptations and metabolic potential of the candidate division KB1 bacteria based on a partial single cell genome. *Front. Microbiol.* 7:1266. 10.3389/fmicb.2016.01266 27597842 PMC4993014

[B57] Pachiadaki M. G. Kormas K. A. (2013). Interconnectivity versus isolation of prokaryotic communities in European deep-sea mud volcanoes. *Biogeosciences* 10 2821–2831. 10.5194/bg-10-2821-2013

[B58] Pachiadaki M. G. Kallionaki A. Shlmann D. De Lange A. Kormas K. J. (2011). Diversity and spatial distribution of prokaryotic communities along a sediment vertical profile of a deep-sea mud volcano. *Microb. Ecol.* 62 655–668. 10.1007/s00248-011-9855-2 21538105

[B59] Pachiadaki M. G. Lykousis V. Stefanou E. G. Kormas K. A. (2010). Prokarotic community structure and diversity in the sediments of an active submarine mud volcano (Kazan mud volcano, East Mediterranean Sea). *FEMS Microbiol. Ecol.* 72 429–444. 10.1111/j.1574-6941.2010.00857.x 20370830

[B60] Pagé A. Juniper S. K. Olagnon M. Alain K. Desrosiers G. Quérellou J. (2004). Microbial diversity associated with a *Paralvinella sulfincola* tube and the adjacent substratum on an active deep-sea vent chimney. *Geobiology* 2 225–238. 10.1111/j.1472-4677.2004.00034.x

[B61] Pikuta E. Lysenko A. Chuvilskaya N. Mendrock U. Hippe H. Suzina N. (2000). *Anoxybacillus pushchinensis* gen. nov., sp. nov., a novel anaerobic, alkaliphilic, moderately thermophilic bacterium from manure, and description of *Anoxybacillus flavithermus* comb. nov. *Int. J. Syst. Evol. Microbiol.* 50 2109–2117. 10.1099/00207713-50-6-2109 11155986

[B62] Pimmel A. Claypool G. (2001). *Introduction to shipboard organic geochemistry on the JOIDES resolution*. ODP Technical Note, 30. College Station TX: Ocean Drilling Program. 10.2973/odp.tn.30.2001

[B63] Pruesse E. Peplies J. Glöckner F. O. (2012). SINA: Accurate high-throughput multiple sequence alignment of ribosomal RNA genes. *Bioinformatics* 28 1823–1829. 10.1093/bioinformatics/bts252 22556368 PMC3389763

[B64] Rattray J. E. Chakraborty A. Elizondo G. Ellefson E. Bernard B. Brooks J. (2022). Endospores associated with deep seabed geofluid features in the eastern Gulf of Mexico. *Geobiology* 20 823–836. 10.1111/gbi.12517 35993193 PMC9804197

[B65] Ruff E. Biddle J. F. Teske A. Knittel K. Boetius A. Ramette A. (2015). Global dispersion and local diversification of the methane seep microbiome. *Proc. Natl. Acad. Sci. U.S.A.* 112 4015–4020. 10.1073/pnas.1421865112 25775520 PMC4386351

[B66] Ryan R. P. Monchy S. Cardinale M. Taghavi S. Crossman L. Avison M. B. (2009). The versatility and adaptation of bacteria from the genus *Stenotrophomonas*. *Nat. Rev. Microbiol.* 7 514–525. 10.1038/nrmicro2163 19528958

[B67] Sass A. McKew B. A. Sass H. Fichtel J. Timmis K. N. McGenity T. J. (2008). Diversity of *Bacillus*-like organisms from deep-sea hypersaline anoxic sediments. *Saline Syst.* 4:8. 10.1186/1746-1448-4-8 18541011 PMC2464584

[B68] Schloss P. D. (2013). Secondary structure improves OTU assignments of 16S rRNA gene sequences. *ISME J.* 7 457–460. 10.1038/ismej.2012.102 23018771 PMC3578559

[B69] Schloss P. D. Handelsman J. (2005). Introducing DOTUR, a computer program for defining operational taxonomic units and estimating species richness. *Appl. Environ. Microbiol.* 71 1501–1506. 10.1128/AEM.71.3.1501-1506.2005 15746353 PMC1065144

[B70] Schloss P. D. Westcott S. L. Ryabin T. Hall J. R. Hartmann M. Hollister E. B. (2009). Introducing mothur: Open-source, platform-independent, community-supported software for describing and comparing microbial communities. *Appl. Environ. Microbiol.* 75 7537–7541. 10.1128/AEM.01541-09 19801464 PMC2786419

[B71] Schmidtova J. Hallam S. J. Baldwin S. A. (2009). Phylogenetic diversity of transition and anoxic zone bacterial communities within a near-shore anoxic basin: Nitinat lake. *Environ. Microbiol.* 11 3233–3251. 10.1111/j.1462-2920.2009.02044.x 19735278

[B72] Scholz C. F. P. Kilian M. (2016). The natural history of cutaneous *Propionibacteria*, and reclassification of selected species within the genus *Propionibacterium* to the proposed novel genera *Acidipropionibacterium* gen. nov., *Cutibacterium* gen. nov., and *Pseudopropioninbacterium* gen. nov. *Int. J. Syst. Evol. Microbiol.* 66 4422–4432. 10.1099/ijsem.0.001367 27488827

[B73] Siam R. Mustafa G. A. Sharaf H. Moustafa A. Ramadan A. R. Antunes A. (2012). Unique prokaryotic consortia in geochemically distinct sediments from Red Sea Atlantis II and discovery deep brine pools. *PLoS One* 7:e42872. 10.1371/journal.pone.0042872 22916172 PMC3423430

[B74] Sørensen K. B. Canfield D. E. Teske A. P. Oren A. (2005). Community composition of a hypersaline endoevaporitic microbial mat. *Appl. Environ. Microbiol.* 71 7352–7365. 10.1128/AEM.71.11.7352-7365.2005 16269778 PMC1287706

[B75] Sorokin D. Y. Messina E. La Cono V. Ferrer M. Ciordia S. Mena M. C. (2018). Sulfur respiration in a group of facultatively anaerobic Natronoarchaea ubiquitous in hypersaline soda lakes. *Front. Microbiol.* 9:02359. 10.3389/fmicb.2018.02359 30333814 PMC6176080

[B76] Sorokin D. Y. Tourova T. P. Panteleeva A. N. Muyzer G. (2012). *Desulfonatronobacter acidivorans* gen. nov., sp. nov. and *Desulfobulbus alkaliphilus* sp. nov., haloalkaliphilic heterotrophic sulfate-reducing bacteria from soda lakes. *Int. J. Syst. Evol. Microbiol.* 62 2107–2113. 10.1099/ijs.0.029777-0 22039002

[B77] Spang A. Saw J. H. Jørgensen S. L. Zaremba-Niedzwiedzka K. Martijn J. Lind A. E. (2015). Complex archaea that bridge the gap between prokaryotes and eukaryotes. *Nature* 521 173–179. 10.1038/nature14447 25945739 PMC4444528

[B78] Stahl D. A. Amann R. (1991). “Development and application of nucleic acid probes in bacterial systematics,” in *Sequencing and hybridization techniques in bacterial systematics*, eds Stackebrandt E. Goodfellow M. (New York, NY: John Wiley & Sons), 205–248.

[B79] Sturt H. F. Summons R. E. Smith K. Elvert M. Hinrichs K. U. (2004). Intact polar membrane lipids in prokaryotes and sediments deciphered by high-performance liquid chromatography/electrospray ionization multistage mass spectrometry–new biomarkers for biogeochemistry and microbial ecology. *Rapid Commun. Mass Spectrom.* 18 617–628. 10.1002/rcm.1378 15052572

[B80] Suess J. Engelen B. Cypionka H. Sass H. (2004). Quantitative analysis of bacterial communities from Mediterranean sapropels based on cultivation-dependent methods. *FEMS Microbiol. Ecol.* 51 109–121. 10.1016/j.femsec.2004.07.010 16329860

[B81] Suess J. Schubert K. Sass H. Cypionka H. Overmann J. Engelen B. (2006). Widespread distribution and high abundance of *Rhizobium radiobacter* within Mediterranean subsurface sediments. *Environ. Microbiol.* 8 1753–1763. 10.1111/j.1462-2920.2006.01058.x 16958756

[B82] Swan B. K. Ehrhardt C. J. Reifel K. M. Moreno L. I. Valentine D. L. (2010). Archaeal and bacterial communities respond differently to environmental gradients in anoxic sediments of a California hypersaline lake, the Salton Sea. *Appl. Environ. Microbiol.* 76 757–768. 10.1128/AEM.02409-09 19948847 PMC2812989

[B83] Swofford D. L. (2000). *PAUP*. Phylogenetic analysis using parsimony (and other methods)*, 4th Edn. Sunderland, MA: Sinauer Associates.

[B84] Teske A. (2019). “Hydrocarbon-degrading anaerobic microbial communities in natural oil seeps,” in *Microbial communities utilizing hydrocarbons and lipids: Members, metagenomics and ecophysiology*, ed. McGenity T. J. (Cham: Springer). 10.1007/978-3-030-14785-3_3

[B85] Teske A. Joye S. B. (2020). “The gulf of mexico: An introductory survey of a seep-dominated seafloor landscape,” in *Marine hydrocarbon seeps–microbiology and biogeochemistry of a global marine habitat*, eds Teske A. Carvalho V. (Berlin: Springer), 69–100. 10.1007/978-3-030-34827-4_4

[B86] Teske A. Hinrichs K. U. Edgcomb V. de Vera Gomez A. Kysela D. Sylva S. P. (2002). Microbial diversity in hydrothermal sediments in the Guaymas Basin: Evidence for anaerobic methanotrophic communities. *Appl. Environ. Microbiol.* 68 1994–2007. 10.1128/AEM.68.4.1994-2007.2002 11916723 PMC123873

[B87] van den Bogert B. Erkus O. Boekhorst J. de Goffau M. Smid E. J. Zoetendal E. G. (2013). Diversity of human small intestinal *Streptococcus* and *Veillonella* populations. *FEMS Microbiol. Ecol.* 85 376–388. 10.1111/1574-6941.12127 23614882

[B88] van der Wielen P. W. J. J. Bolhuis H. Borin S. Daffonchio D. Corselli C. Giuliano L. (2005). The enigma of prokaryotic life in deep hypersaline anoxic basins. *Science* 307 121–123. 10.1126/science.1103569 15637281

[B89] Vandamme P. Coenye T. (2004). Taxonomy of the genus *Cupriavidus*: A tale of lost and found. *Int. J. Syst. Evol. Microbiol.* 54 2285–2289. 10.1099/ijs.0.63247-0 15545472

[B90] Vetriani C. Jannasch H. W. MacGregor B. J. Stahl D. A. Reysenbach A. L. (1999). Population structure and phylogenetic characterization of marine benthic archaea in deep-sea sediments. *Appl. Environ. Microbiol.* 65 4375–4384. 10.1128/AEM.65.10.4375-4384.1999 10508063 PMC91581

[B91] Vreeland R. H. Litchfield C. D. Martin E. L. Elliot E. (1980). *Halomonas elongata*, a new genus and species of extremely salt-tolerant bacteria. *Int. J. Syst. Bacteriol.* 30 485–495. 10.1007/s11274-012-1020-7 22806037

[B92] Wang B. Lai Q. Cui Z. Tan T. Shao Z. (2008). A pyrene-degrading consortium from deep-sea sediment of the West Pacific and its key member *Cycloclasticus* sp. P1. *Environ. Microbiol.* 10 1948–1963. 10.1111/j.1462-2920.2008.01611.x 18430013

[B93] Wang L. Shao Z. (2021). Aerobic denitrification and heterotrophic sulfur oxidation in the genus *Halomonas* revealed by six novel species characterizations and genome-based analysis. *Front. Microbiol.* 12:652766. 10.3389/fmicb.2021.652766 33815342 PMC8014003

[B94] Wen A. Fegan M. Hayward C. Chakraborty S. Sly L. I. (1999). Phylogenetic relationships among members of the Comamonadaceae and description of *Delftia acidovorans* (den Dooren de Jong 1926 and Tamaoka et al 1987) gen. nov., comb. nov. *Int. J. Syst. Microbiol.* 49 567–576. 10.1099/00207713-49-2-567 10319477

[B95] Westbrook G. K. Reston T. J. (2002). The accretionary complex of the Mediterranean Ridge: Tectonics, fluid flow and the formation of brine lakes. *Mar. Geol.* 186 1–8. 10.1016/S0025-3227(02)00169-X

[B96] Wirsen C. O. Sievert S. M. Cavanaugh C. M. Molyneaux S. J. Ahmad A. Taylor L. T. (2002). Characterization of an autotrophic sulfide-oxidizing marine *Arcobacter* sp. that produces filamentous sulfur. *Appl. Environ. Microbiol.* 68 316–325. 10.1128/AEM.68.1.316-325.2002 11772641 PMC126556

[B97] Xiang X. Wang R. Wang H. Ging L. Man B. Xu Y. (2016). Distribution of Bathyarchaeota communities across different terrestrial settings and their potential ecological functions. *Sci. Rep.* 7:45028. 10.1038/srep45028 28322330 PMC5359579

[B98] Yabuuchi E. Yano I. (1981). *Achromobacter* gen. nov. and *Achromobacter xylosoxidans* (ex Yabuuchi and Ohyama 1971) nom. rev. *Int. J. Syst. Bacteriol.* 31 477–478. 10.1099/00207713-31-4-477

[B99] Yakimov M. M. Giuliano L. Cappello S. Denaro R. Golyshin P. N. (2007). Microbial community of a hydrothermal mud vent underneath the deep-sea anoxic brine lake Urania (Eastern Mediterranean). *Orig. Life Evol. Biosph.* 37 177–188. 10.1007/s11084-006-9021-x 17136435

[B100] Yakimov M. M. La Cono V. Slepak V. Z. La Spada G. Arcadi E. Messina E. (2013). Microbial life in the lake Medeé, the largest deep-sea salt-saturated formation. *Sci. Rep.* 3:3554. 10.1038/srep03554 24352146 PMC3867751

[B101] You Z.-Q. Li J. Qin S. Tian X.-P. Wang F.-Z. Zhang S. (2013). *Bacillus abyssalis* sp. nov., isolated from a sediment of the South China Sea. *Antonie Van Leeuwenhoek* 103 963–969. 10.1007/s10482-013-9875-7 23314911

[B102] Youssef N. H. Farag I. F. Rudy S. Mulliner A. Walker K. Caldwell F. (2019). The wood-Ljungdahl pathway as a key component of metabolic versatility in Candidate phylum Bipolaricaulota (Acetothermia, OP1). *Environ. Microbiol. Rep.* 11 538–547. 10.1111/1758-2229.12753 30888727

[B103] Zabel M. (2012). RV METEOR, cruise report M84/L1. Biogeochemistry and methane hydrates of the black sea, oceanography of the Mediterranean, shelf sedimentation and cold water carbonates. *DFG Senatskommission Ozeanogr.* 39 1–38.

[B104] Zhao W. Zhang C. L. Romanek C. S. Wiegel J. (2008). Description of *Caldalkalibacillus uzonensis* sp. nov. and emended description of the genus *Caldalkalibacillus*. *Int. J. Syst. Evol. Microbiol.* 58 1106–1108. 10.1099/ijs.0.65363-0 18450697

[B105] Zhou Z. Liu Y. Lloyd K. G. Pan J. Yang Y. Gu J.-D. (2019). Genomic and transcriptomic insights into the ecology and metabolism of benthic archaeal cosmopolitan, Thermoprofundales (MBG-D archaea). *ISME J.* 13 885–901. 10.1038/s41396-018-0321-8 30514872 PMC6461988

