# Supplementary material for: Microbial diversity gradients in the geothermal mud volcano underlying the hypersaline Urania Basin
Source: Front Microbiol. 2022 Dec 21;13:1043414. doi: 10.3389/fmicb.2022.1043414 (PMC9812581; doi:10.3389/fmicb.2022.1043414)
Supplement: Supplementary Table 1 — Concentration and 13C stable isotope values of methane and ethane in Urania Basin brine, fluid mud, and subsurface sediment samples. [file Table_1.DOCX]

**Table S1**.

| **GeoB** | **Type** | **Sample Depth** | |  | **Porosity** | **Methane** | **Ethane** | **C1/C2** | **d13C Methane** | **d13C Methane** | **d13C Ethane** | **d13C Ethane** |
| --- | --- | --- | --- | --- | --- | --- | --- | --- | --- | --- | --- | --- |
|  |  | **top** | **bottom** | **mean** |  |  |  |  | **mean** | **SD** | **mean** | **SD** |
|  |  | **[mbsf]** | **[mbsf]** | **[mbsf]** |  | **[µM]** | **[µM]** |  | **[permil vs VPDB]** | **[permil vs VPDB]** | **[permil vs VPDB]** | **[permil vs VPDB]** |
|  |  |  |  |  |  |  |  |  |  |  |  |  |
| 15101-4 | MUC | 0,06 | 0,09 | 0,075 | 0,51 | 1370 | 242 | 5,7 | -29,9 | 0,2 | -29,4 | 0,1 |
| 15101-4 | MUC | 0,35 | 0,38 | 0,365 | 0,64 | 920 | 173 | 5,3 | -30,5 | 0,0 | -29,5 | 0,0 |
|  |  |  |  |  |  |  |  |  |  |  |  |  |
| 15101-7 | Gravity core | 0,00 | 0,04 | 0,02 | 0,69 | 1110 | 216 | 5,1 | -30,9 | 0,2 | -29,5 | 0,0 |
| 15101-7 | Gravity core | 0,27 | 0,30 | 0,29 | 0,82 | 432 | 106 | 4,0 | -30,5 | 0,2 | -29,3 | 0,1 |
| 15101-7 | Gravity core | 0,50 | 0,54 | 0,52 | 0,75 | 1260 | 231 | 5,5 | -30,9 | 0,0 | -29,5 | 0,0 |
| 15101-7 | Gravity core | 0,59 | 0,61 | 0,60 | 0,70 | 1330 | 254 | 5,3 | -30,5 | 0,1 | -29,1 | 0,1 |
| 15101-7 | Gravity core | 1,10 | 1,13 | 1,12 | 0,70 | 1290 | 243 | 5,3 | -30,9 | 0,4 | -29,5 | 0,1 |
| 15101-7 | Gravity core | 1,50 | 1,54 | 1,52 | 0,53 | 1450 | 264 | 5,5 | -30,3 | 0,1 | -29,3 | 0,1 |
| 15101-7 | Gravity core | 1,60 | 1,62 | 1,61 | 0,59 | 2650 | 489 | 5,4 | -29,9 | 0,0 | -29,1 | 0,1 |
| 15101-7 | Gravity core | 2,12 | 2,16 | 2,14 | 0,65 | 421 | 95 | 4,4 | -30,5 | 0,0 | -28,8 | 0,1 |
| 15101-7 | Gravity core | 2,46 | 2,50 | 2,48 | 0,59 | 1150 | 214 | 5,4 | -31,1 | 0,0 | -29,6 | 0,1 |
| 15101-7 | Gravity core | 2,61 | 2,63 | 2,62 | 0,58 | 1240 | 243 | 5,1 | -31,0 | 0,1 | -29,2 | 0,1 |
| 15101-7 | Gravity core | 3,08 | 3,11 | 3,10 | 0,57 | 1240 | 267 | 4,7 | -30,8 | 0,4 | -29,3 | 0,0 |
| 15101-7 | Gravity core | 3,46 | 3,50 | 3,48 | 0,55 | 3330 | 599 | 5,6 | -30,7 | 0,2 | -29,6 | 0,0 |
| 15101-7 | Gravity core | 3,61 | 3,65 | 3,63 | 0,56 | 289 | 70 | 4,1 | -27,8 | 0,0 | -27,3 | 0,1 |
| 15101-7 | Gravity core | 4,28 | 4,31 | 4,30 | 0,49 | 574 | 143 | 4,0 | -29,6 | 0,1 | -27,4 | 0,1 |
| 15101-7 | Gravity core | 4,46 | 4,50 | 4,48 | 0,43 | 670 | 255 | 2,6 | -29,7 | 0,0 | -29,2 | 0,0 |
|  |  |  |  |  |  |  |  | 4,9 | -30,3 |  | -29,1 |  |

| **GeoB** | **Type** | **Sample Depth** | |  |  | **Methane** | **Ethane** | **C1/C2** | **d13C Methane** | **d13C Methane** | **d13C Ethane** | **d13C Ethane** |
| --- | --- | --- | --- | --- | --- | --- | --- | --- | --- | --- | --- | --- |
|  |  | **Depth** | **Rope Length** | **Depth above seafloor** | **Sample #** |  |  |  | **mean** | **SD** | **mean** | **SD** |
|  |  | **[dbar]** | **[m]** | **[m]** |  | **[µM]** | **[µM]** |  | **[permil vs VPDB]** | **[permil vs VPDB]** | **[permil vs VPDB]** | **[permil vs VPDB]** |
|  |  |  |  |  |  |  |  |  |  |  |  |  |
| 15101-6 | CTD | 3022 | 3000 | -750 | 1 | 0,5 | 0,05 | 10,0 | BD |  | BD |  |
| 15101-6 | CTD | 3633 | 3560 | -190 | 2 | 530 | 95 | 5,6 | NA |  | NA |  |
| 15101-6 | CTD | 3645 | 3570 | -180 | 3 | 778 | 132 | 5,9 | -31,0 | 0,1 | -29,4 | 0,1 |
| 15101-6 | CTD | 3656 | 3580 | -170 | 4 | 415 | 73 | 5,7 | -30,7 |  | -30,3 |  |
| 15101-6 | CTD | 3667 | 3590 | -160 | 5 | 555 | 95 | 5,8 | NA |  | NA |  |
| 15101-6 | CTD | 3677 | 3600 | -150 | 6 | 466 | 96 | 4,9 | -30,5 | 0,6 | -30,7 | 0,1 |
| 15101-6 | CTD | 3677 | 3603 | -147 | 24 | 661 | 113 | 5,8 | -27,3 | 0,3 | -28,8 |  |
| 15101-6 | CTD | 3685 | 3605 | -145 | 7 | 442 | 79 | 5,6 | -30,4 |  | -30,3 |  |
| 15101-6 | CTD | 3681 | 3607 | -143 | 23 | 449 | 78 | 5,7 | -30,6 |  | -29,8 |  |
| 15101-6 | CTD | 3687 | 3610 | -140 | 8 | 332 | 88 | 3,8 | -32,0 |  | -30,8 |  |
| 15101-6 | CTD | 3692 | 3615 | -135 | 22 | 280 | 60 | 4,7 | NA |  | NA |  |
| 15101-6 | CTD | 3702 | 3620 | -130 | 9 | 285 | 64 | 4,5 | -31,7 |  | -30,4 |  |
| 15101-6 | CTD | 3717 | 3630 | -120 | 10 | 287 | 53 | 5,4 | -31,5 | 0,1 | -31,0 | 1,0 |
| 15101-6 | CTD | 3732 | 3640 | -110 | 11 | 562 | 108 | 5,2 | -31,2 |  | -30,3 |  |
| 15101-6 | CTD | 3747 | 3650 | -100 | 12 | 566 | 101 | 5,6 | NA |  | NA |  |
| 15101-6 | CTD | 3762 | 3660 | -90 | 13 | 792 | 128 | 6,1 | NA |  | NA |  |
| 15101-6 | CTD | 3776 | 3670 | -80 | 14 | 362 | 70 | 5,2 | -30,8 |  | -29,9 |  |
| 15101-6 | CTD | 3797 | 3680 | -70 | 15 | 176 | 34 | 5,1 | NA |  | NA |  |
| 15101-6 | CTD | 3807 | 3690 | -60 | 16 | 141 | 26 | 5,4 | NA |  | NA |  |
| 15101-6 | CTD | 3827 | 3700 | -50 | 17 | 539 | 103 | 5,2 | -31,0 |  | -30,0 |  |
| 15101-6 | CTD | 3836 | 3710 | -40 | 18 | 169 | 32 | 5,3 | NA |  | NA |  |
| 15101-6 | CTD | 3851 | 3720 | -30 | 19 | 413 | 79 | 5,2 | NA |  | NA |  |
| 15101-6 | CTD | 3867 | 3730 | -20 | 20 | 437 | 83 | 5,3 | NA |  | NA |  |
| 15101-6 | CTD | 3875 | 3736 | -14 | 21 | 456 | 79 | 5,8 | NA |  | NA |  |
|  |  |  |  |  |  |  |  | 5,3 | -30,7 |  | -30,2 |  |

BD = below detection limit for stable carbon isotope analysis

NA = not analyzed because of sample loss during transport
