# Supplementary material for: Microbial diversity gradients in the geothermal mud volcano underlying the hypersaline Urania Basin
Source: Front Microbiol. 2022 Dec 21;13:1043414. doi: 10.3389/fmicb.2022.1043414 (PMC9812581; doi:10.3389/fmicb.2022.1043414)
Supplement: Supplementary Table 2 — Concentrations of hydrocarbons in the subseafloor of the Urania Basin. PAH/HC, portion of polyaromatic hydrocarbons relative to sum of all identified hydrocarbons; Thermal HC/HC, portion of thermally altered hydrocarbons relative to sum of all identified hydrocarbons. [file Table_2.DOCX]

**Table S2**.

| Compound | GeoB15101-7 10-30 cm | | GeoB15101-7 260-280 cm | |
| --- | --- | --- | --- | --- |
|  | ng/g Sed | µg/TOC | ng/g Sed | µg/TOC |
| Trimethylnaphthalene | 3.34 | 11.12 | 0.89 | 2.97 |
| Fluorene | 10.66 | 35.52 | 2.13 | 7.11 |
| 4-Methyl-C_16_ alkane | 9.52 | 31.75 | 5.47 | 18.25 |
| Methylfluorene | 11.50 | 38.32 | 6.16 | 20.53 |
| Benzidine | 15.68 | 52.26 | 9.74 | 32.48 |
| Phenanthrene | 24.98 | 83.28 | 16.21 | 54.03 |
| Pristane | 3.26 | 10.88 | 2.67 | 8.91 |
| Methylphenanthrene | 5.13 | 17.11 | 4.20 | 13.98 |
| unidentified triterpane | 3.81 | 12.71 | 3.00 | 9.99 |
| C_21_-alkane | 3.18 | 10.60 | 2.92 | 9.72 |
| Pyrene | 12.65 | 42.16 | 11.05 | 36.82 |
| Pyrene+ | 6.29 | 20.96 | 6.24 | 20.80 |
| PMI | 3.70 | 12.34 | 3.25 | 10.85 |
| C_23_-alkane | 1.41 | 4.71 | 1.25 | 4.16 |
| C_25_-alkane | 1.51 | 5.04 | 1.19 | 3.96 |
| Squalane | 5.55 | 18.50 | 5.16 | 17.21 |
| C_27_-alkane | 1.35 | 4.50 | 1.15 | 3.83 |
| Branched unsaturated C_27_-alkene | 4.53 | 15.09 | 4.13 | 13.76 |
| C_29_-alkane | 1.22 | 4.08 | 1.13 | 3.75 |
| Hop17,21-ene | 2.22 | 7.38 | 1.95 | 6.51 |
| C_31_-alkane | 0.77 | 2.58 | 0.81 | 2.71 |
| C_33_-alkane | 0.15 | 0.51 | 0.23 | 0.78 |
| Lycopane | 1.86 | 6.22 | 1.61 | 5.37 |
| PAH/HC (%) |  | 67.2 |  | 61.2 |
| Thermal/HC (%) |  | 86.7 |  | 82.6 |
