# Supplementary material for: Microbial diversity gradients in the geothermal mud volcano underlying the hypersaline Urania Basin
Source: Front Microbiol. 2022 Dec 21;13:1043414. doi: 10.3389/fmicb.2022.1043414 (PMC9812581; doi:10.3389/fmicb.2022.1043414)
Supplement: Supplementary Table 3 — Similarities of Urania Basin phylotypes and related sequences of pure cultures and environmental phylotypes. Genbank numbers for bacteria and archaea are: OP389385-OP389468 and OP389469-OP389561 in sample 5; OP352680-OP352749 and OP389299-OP389384 in sample 8; OP352780-OP389844 and OP389717-OP389809 in sample 14; OP389562-OP389631 and OP389897-OP389989 in sample 21; OP352539-OP352606, OP389632-OP389716, and OP856603-OP856618 in sample GC-10-30; OP352609-OP352677, OP389811-OP389896, and OP856619-OP856629 for sample GC-260-280. [file Table_3.docx]

| Urania Basin sequences | Number of near-identical clones | Genbank number | % similarity to phylogeny sequence | related Genbank sequence, used in phylogeny | Phylogenetic affiliation to cultured bacteria and archaea, or major uncultured group |
| --- | --- | --- | --- | --- | --- |
| Brine 05-14  Bacteria | 4 | OP389394 | 98.6% | DQ521799  Hypersaline Gulf of Mexico seep | *Desulfatiglans* *anilini*  (Deltaproteobacteria) |
| Brine 05-48  Bacteria | 1 | OP389427 | 92.3% | NR117486  Pure culture | *Desulfonatronobacter* *acidivorans*  (Deltaproteobacteria) |
| Brine 05-81  Bacteria | 1 | OP389455 | 94.7% | AJ937690  Terrestrial Mud volcano | *Desulfococcus* *oleovorans*  (Deltaproteobacteria) |
| Brine 05-40  Bacteria | 1 | OP389419 | 76.7% | AJ347756  Shaban Deep brine interface | *Desulfobulbus* *mediterraneus*  (Deltaproteobacteria) |
| Brine 05-42  Bacteria | 2 | OP389421 | 97.3% | AF420352  Guaymas Basin sed. | *Sulfurimonas autotrophicum*  (Epsilonproteobacteria) |
| Brine 05-15  Bacteria | 2 | OP389395 | 95.5% | NR024802  Pure culture | *Sulfurovum lithotrophicum*  (Epsilonproteobacteria) |
| Brine 05-27  Bacteria | 4 | OP389407 | 97.4% | AM268257  Urania Basin Mud volcano | *Arcobacter sulfidicus*  (Epsilonproteobacteria) |
| Brine 05-25  Bacteria | 10 | OP389405 | 79.7% | FJ712527  Kazan Mud Volcano | MSBL5  (Chloroflexi) |
| Brine 05-64  Bacteria | 1 | OP389442 | 98.7% | JN442690 hypersaline Microbial Mat | *Anaerohalosphaera* *lusitana*  (Planctomycetes) |
| Brine 05-28  Bacteria | 2 | OP389408 | 99.7% | AY547876  Urania Basin brine interface | OD1 lineage  (Parcubacteria) |
| Brine 05-67  Bacteria | 3 | OP389445 | 98.5% | KR857668  Orca Basin hyper-saline sediment | MSBL2  (not subsumed under another phylum) |
| Brine 05-49  Bacteria | 1 | OP389428 | 99.4% | AY547927  Urania Basin brine interface | MSBL2 sister lineage |
| Brine 05-56  Bacteria | 1 | OP389434 | 99.2% | EF687321  Chefren Mud volcano | Cytophagales  (Bacteroidetes) |
| Brine 05-08  Bacteria | 2 | OP389390 |  | AJ133617  Kebrit Deep brine | KB1 Type I |
| Brine 05-03  Bacteria | 47 | OP389387 | 94% | DQ521797  Hypersaline Gulf of Mexico seep | KB1 Type II |
| Brine 05-02  Archaea | 9 | OP389470 | 98.9% | AY226377  Urania Basin brine | MSBL-1 archaea  (Euryarchaeota) |
| Brine 05-33  Archaea | 4 | OP389500 | 97% | AM268267  Urania Basin mud volcano | MSBL-1 archaea, paraphyletic lineage  (Euryarchaeota) |
| Brine 05-22  Archaea | 1 | OP389489 | 95.9% | AB177019  Peru Margin subsurface | Marine Benthic Group D  (Thermoplasmatales) |
| Brine 05-50  Archaea | 79 | OP389517 | 99.7% | HE604632  Hypersaline lake Kazin sediment | *Halodesulfoarchaeum* *formicicum*  (Halobacteria) |
| Fluid Mud 08-09 Bacteria | 6 | OP352691 | 98.8% | NR_119220  Pure culture | *Stenotrophomonas* *maltophila*  (Gammaproteobacteria) |
| Fluid Mud 08-46 Bacteria | 3 | OP352718 | 99% | NR_027607  Pure culture | *Cupriavidus* *metallidurans*  (Betaproteobacteria) |
| Fluid Mud 08-03 Bacteria | 4 | OP352687 | 99.4% | CP053381  Pure culture | *Halomonas* *sulfidoxydans*  (Gammaproteobacteria) |
| Fluid Mud 08-05 Bacteria | 4 | OP352688 | 99.8% | NR_025900  Pure culture | *Thermus* *aquaticus*  (Thermales) |
| Fluid Mud 08-33 Bacteria | 7 | OP352708 | 98.7% | NR_109671  Pure culture | *Bacillus* *abyssalis*  (Firmicutes) |
| Fluid Mud 08-06 Bacteria | 12 | OP352689 | 95.8% | NR_116916  Pure culture | *Piscibacillus halophilius*  (Firmicutes) |
| Fluid Mud 08-14 Bacteria | 3 | OP352695 | 99.1% | NR_026516  Pure culture | *Anoxybacillus* *flavithermus*  (Firmicutes) |
| Fluid Mud 08-15 Bacteria | 12 | OP352696 | 97.8% | NR_151896  Pure culture | *Anoxybacillus* *geothermalis*  (Firmicutes) |
| Fluid Mud 08-01 Bacteria | 7 | OP352686 | 99.7% | GU936608  Pure culture | *Geobacillus pallidus*  (Firmicutes) |
| Fluid Mud 08-01 Archaea | 63 | OP389299 | 99.5% | AY226377  Urania Basin brine | MSBL-1  (Euryarchaeota) |
| Fluid Mud 08-33 Archaea | 4 | OP389329 | 85% | AM268267  Urania Basin mud volcano | MSBL-1 sister lineage  (Euryarchaeota) |
| Fluid Mud 08-14 Archaea | 19 | OP389311 | 99.4% | NR_149760  Pure culture | *Halodesulfoarchaeum formicicum* (Halobacteria) |
| Fluid Mud 08-23 (08-82) Archaea | 4 | OP389320  (OP389373) | 95.4%  (90.2%) | AB801148  Shimokita subsurface | Bathyarchaeota/MCG17 |
| Fluid Mud 14-06 Bacteria | 1 | OP352780 | 99.2% | NR_036865  Pure culture | *Bradyrhizobium japonicum*  (Alphaproteobacteria) |
| Fluid Mud 14-01 Bacteria | 12 | OP352796 | 98.9% | NR_119220  Pure culture | *Stenotrophomonas maltophila* (Gammaproteobacteria) |
| Fluid Mud 14-05 Bacteria | 3 | OP352800 | 99.1% | NR_027607  Pure culture | *Cupriavidus metallidurans*  (Betaproteobacteria) |
| Fluid Mud 14-09 Bacteria | 4 | OP352781 | 99.4% | CP053381  Pure culture | *Halomonas sulfidoxydans*  (Gammaproteobacteria) |
| Fluid Mud 14-87 Bacteria | 1 | OP352839 | 92.1% | FJ712527  Kazan Mud volcano | Chloroflexi |
| Fluid Mud 14-33 Bacteria | 1 | OP352783 | 99.9% | AY735097  Pure culture | *Spirochaeta* *xylanolyticus*  (Spirochaetes) |
| Fluid Mud 14-10 Bacteria | 7 | OP352802 | 98.7% | NR109671  Pure culture | *Bacillus abyssalis*  (Firmicutes) |
| Fluid Mud 14-36 Bacteria | 1 | OP352784 | 99% | NR_148762  Pure culture | *Terrilactibacillus laevilacticus*  (Firmicutes) |
| Fluid Mud 14-39 Bacteria | 2 | OP352818 | 99.4% | NR026516  Pure culture | *Anoxybacillus flavithermus*  (Firmicutes) |
| Fluid Mud 14-18 Bacteria | 16 | OP352805 | 97.7% | NR151896  Pure culture | *Anoxybacillus geothermalis*  (Firmicutes) |
| Fluid Mud 14-02 Bacteria | 6 | OP352797 | 99.6% | GU936608  Pure culture | *Geobacillus pallidus*  (Firmicutes) |
| Fluid Mud 14-63 Bacteria | 1 | OP352789 | 98.9% | FN428694  Pure culture | *Geobacillus stearotherophilus*  (Firmicutes) |
| Fluid Mud 14-47 Bacteria | 7 | OP352786 | 99.7% | JN230180  Marine Subsurface | Uncultured Firmicutes  (Firmicutes) |
| Fluid Mud 14-03 Archaea | 32 | OP389719 | 99.5% | AY226377  Urania Basin brine | MSBL1  (Euryarchaeota) |
| Fluid Mud 14-62 Archaea | 62 | OP389777 | 93.2% | AB177019 Peru Margin Subsurface | Marine Benthic Group D  (Thermoplasmatales) |
| Fluid Mud 14-25 Archaea | 1 | OP389741 | 97.9% | AB644523  Shimokita subsurface | Marine Thermoplasmata  (Thermoplasmatales) |
| Fluid Mud 21-39 Bacteria | 4 | OP389563 | 99% | NR_036865  Pure culture | *Bradyrhizobium japonicum*  (Alphaproteobacteria) |
| Fluid Mud 21-08 Bacteria | 6 | OP389575 | 98.6% | NR_119220  Pure culture | *Stenotrophomonas maltophila*  (Gammaproteobacteria) |
| Fluid Mud 21-83  Bacteria | 1 | OP389626 | 99% | NR_027607  Pure culture | *Cupriavidus metallidurans*  (Betaproteobacteria) |
| Fluid Mud 21-37 Bacteria | 2 | OP389562 | 99% | AM268241  Urania Basin mud pit | *Ca*. Acetothermus autotrophicum (Acetothermia) |
| Fluid Mud 21-07 Bacteria | 1 | OP389574 | 99.8% | NR_025900  Pure culture | *Thermus aquaticus*  (Thermales) |
| Fluid Mud 21-26 Bacteria | 12 | OP389591 | 99.2% | NR_026516  Pure culture | *Anoxybacillus flavithermus*  (Firmicutes) |
| Fluid Mud 21-05 Bacteria | 24 | OP389572 | 98.4% | NR_151896  Pure culture | *Anoxybacillus geothermalis*  (Firmicutes) |
| Fluid Mud 21-13 Bacteria | 5 | OP389580 | 99.6% | GU936608  Pure culture | *Geobacillus pallidus*  (Firmicutes) |
| Fluid Mud 21-92 Bacteria | 1 | OP389630 | 96.9% | NR_043653  Pure culture | *Caldialkalibacillus uzonensis*  (Firmicutes) |
| Fluid Mud 21-64 Archaea | 2 | OP389959 | 98.9% | AY226377  Urania Basin brine | MSBL1  (Euryarchaeota) |
| Fluid Mud 21-37 Archaea | 1 | OP389932 | 99.1% | NR_149760  Pure culture | *Halodesulfoarchaeum*  *formicicum*  (Halobacteria) |
| Fluid Mud 21-21 Archaea | 90 | OP389916 | 98.2% | HQ700678  subsurface biofilm | Marine Thermoplasmata lineage |
| Sediment 10-16 bacteria | 16 | OP856603 | 95.2% | NR_025955  Pure culture | *Cycloclasticus* *pugetii*  (Gammaproteobacteria) |
| Sediment 10-21  Bacteria | 25 | OP352557 | 99.7% | JN230180  Marine subsurface | Uncultured Firmicutes  (Firmicutes) |
| Sediment 10-01  Bacteria | 39 | OP352539 | 96.1% | NR_113376  Pure culture | *Veillonella* *criceti*  (Firmicutes) |
| Sediment 10-01  Archaea | 85 | OP389632 | 92.9% | HM244181  Honghu sediment | Urania Basin Euryarchaeotal Group (UBEG) |
| Sediment 260-01 bacteria | 12 | OP352609 | 99.2% | NR_119220  Pure culture | *Stenotrophomonas* *maltophila* (Gammaproteobacteria) |
| Sediment 260-06 bacteria | 18 | OP352613 | 99.1% | NR_024711  Pure culture | *Delftia* *acidovorans*  (Betaproteobacteria) |
| Sediment 260-17 bacteria | 7 | OP352619 | 97.2% | NR_042387  Pure culture | *Acinetobacter* *calcoaceticus*  (Gammaproteobacteria) |
| Sediment 260-03 bacteria | 5 | OP856619 | 98.6% | Y14908  Pure culture | *Achromobacter* *xylosoxidans*  (Betaproteobacteria) |
| Sediment 260-04 bacteria | 27 | OP352611 | 99.7% | JN230180  Marine subsurface | Uncultured Firmicutes  (Firmicutes) |
| Sediment 260-08 bacteria | 6 | OP856620 | 99.3% | AB573714  Pure culture | *Propionibacterium* *acnes*  (Actinobacteria) |
| Sediment 260-10 Archaea | 38 | OP389819 | 96.9% | AB177019 Peru Margin subsurface | Marine Benthic Group D  (Thermoplasmatales) |
| Sediment 260-25 Archaea | 20 | OP389834 | 94% | AB177095 Peru Margin subsurface | Bathyarchaeota/MCG-8 |
| Sediment 260-11 Archaea | 10 | OP389820 | 94.5% | AB801145  Shimokita subsurface | Bathyarchaeota/MCG-6 |
| Sediment 260-17 Archaea | 5 | OP389826 | 98.2% | AB177284 Peru Margin subsurface | *Prometheoarchaeum* *syntrophicum* (Lokiarchaeota) |
