# Supplementary material for: Microbial diversity gradients in the geothermal mud volcano underlying the hypersaline Urania Basin
Source: Front Microbiol. 2022 Dec 21;13:1043414. doi: 10.3389/fmicb.2022.1043414 (PMC9812581; doi:10.3389/fmicb.2022.1043414)
Supplement: Supplementary Data Sheet 1 — Supplementary methods. [file Data_Sheet_1.docx]

**SUPPLEMENTARY METHODS**

## CTD sampling

A CTD cast is a deployment of a CTD (Conductivity-Temperature-Depth) sensor with Niskin bottles, usually arrayed in a circle around the central sensor unit. The CTD/Niskin array is lowered from the ship on a cable to maximum depth, and then pulled up in predetermined increments; at each stop a Niskin bottle is triggered and collects a water sample, or in our case a fluid mud or brine sample. The CTD sensor measures conductivity (as a proxy for salinity) temperature, and depth (via pressure) continually during each cast. Further details of CTD deployment and sampling procedure in Urania Basin are outlined in Aiello et al. 2020.

## Hydrocarbon measurements

Briefly, 2-3 mL of a brine, mud fluid and subseafloor sediment sample was enclosed in a gas-tight 22 ml glass vial with a Teflon septum and heated for 20 min at 60°C. After heating, 100-500 μL of headspace gas were taken with a gas-tight syringe and analyzed on board by gas chromatography-flame ionization detection (GC-FID, Thermo Finnigan Trace GC) using an Alltech AT-Q column (30 m length; 0.32 mm internal diameter), helium as carrier gas (flow rate 3.0 ml min^-1^) and an injector temperature of 200°C. Injection was in split mode and the GC temperature was held for 3 min at 40°C followed by an increase to 120°C at a rate of 10°C min^-1^. The GC-FID was calibrated on a daily basis using hydrocarbon gas standards of different concentrations (Scotty Gases, Air Liquide). Based on volume and partial pressure in the headspace, the total amount of methane and ethane was quantified and normalized to the volume of the sample. For sediment samples, this was corrected by the corresponding porosity of the solid phase sample. For water column samples, the porosity was set to 1. A second set of subsamples was stored in gas-tight 22-ml glass vials with 5 mL NaOH at 4°C for shore-based stable carbon isotope analysis of methane and ethane. These measurements were performed by split-mode injection into a GC (Thermo Finnigan Trace GC) equipped with a Supelco Carboxen-1006 PLOT fused-silica capillary column (30 m length; 0.32 mm internal diameter) coupled to isotope ratio mass spectrometry (IRMS, Finnigan Delta plus XP) via a GC-combustion interface III. Separation of compounds was achieved isothermally at a column temperature of 40°C.

Lipid biomarker extraction was based on a modified Bligh and Dyer protocol (Sturt et al., 2004) to which two more extraction steps with DCM:MeOH (5:1, v/v) were added. Combined organic extracts were blown down to dryness and an aliquot of the TLE was used to retrieve a hydrocarbon fraction by elution with a mixture of n-hexane and dichloromethane (95/5, v/v) on an aminopropyl cartridge (Supelco LC-NH_2_, 500 mg sorbent). Hydrocarbons were analyzed by GC-FID (Thermo Finnigan Trace GC) for quantification and GC-MS (Thermo Finnigan Trace GC coupled to a Trace MS) for identification. Prior to analysis tetracontane was added as injection standard. The GC of both systems was equipped with a Restek 30-m Rxi-5MS fused silica capillary column (0.25 mm i.d., 0.25 µm film thickness) and helium was used as carrier gas (flow rate 1.0 ml min^-1^). The hydrocarbon fractions were injected in splitless mode at 300°C. After 1 min, the GC temperature was raised from 60°C to 150°C with a rate of 10 °C min^-1^, followed by a rate of 4°C min^-1^ up to 310 °C (final hold time: 25 min).

**REFERENCES**

Aiello, I. W., Beaufort, L., Goldhammer, T., Heuer, V. B., Hinrichs, K.-U., and Zabel, M. (2020). Anatomy of a ‘suspended’ seafloor in the dense brine waters of the deep hypersaline Urania basin. Deep Sea Res. II 171:104626. doi: 10.1016/j.dsr2.2019.07.014

Sturt, H.F., Summons, R.E., Smith, K., Elvert, M., and Hinrichs, K.U. (2004). Intact polar membrane lipids in prokaryotes and sediments deciphered by high-performance liquid chromatography/ electrospray ionization multistage mass spectrometry--new biomarkers for biogeochemistry and microbial ecology. Rapid Commun. Mass Spectrom. 18(6), 617-628. doi: 10.1002/rcm.1378.
